# Genome-Wide Identification and Characterization of the NAC Transcription Factor Family in *Musa Acuminata* and Expression Analysis during Fruit Ripening

**DOI:** 10.3390/ijms21020634

**Published:** 2020-01-18

**Authors:** Bin Li, Ruiyi Fan, Qiaosong Yang, Chunhua Hu, Ou Sheng, Guiming Deng, Tao Dong, Chunyu Li, Xinxiang Peng, Fangcheng Bi, Ganjun Yi

**Affiliations:** 1 State Key Laboratory for Conservation and Utilization of Subtropical Agro-bioresources, College of Life Sciences, South China Agricultural University, Guangzhou 510642, China; binxiuke2343@126.com (B.L.); xpeng@scau.edu.cn (X.P.); 2 Key Laboratory of South Subtropical Fruit Biology and Genetic Resource Utilization(MOA), Institute of Fruit Tree Research, Guangdong Academy of Agricultural Sciences, Guangzhou 510640, China; fanruiyi@outlook.com (R.F.); yangqiaosong@gdaas.cn (Q.Y.); huchunhua@gdaas.cn (C.H.); shengou@gdaas.cn (O.S.); dengguiming@gdaas.cn (G.D.); dongtao@gdaas.cn (T.D.); lichunyu@gdaas.cn (C.L.); 3 Guangdong Province Key Laboratory of Tropical and Subtropical Fruit Tree Research, Institute of Fruit Tree Research, Guangdong Academy of Agricultural Sciences, Guangzhou 510640, China

**Keywords:** *Musa accuminata*, NAC gene family, transcription factor, fruit ripening, genome-wide analysis, banana

## Abstract

Banana (*Musa acuminata*, AAA group) is a representative climacteric fruit with essential nutrients and pleasant flavors. Control of its ripening determines both the fruit quality and the shelf life. NAC (NAM, ATAF, CUC2) proteins, as one of the largest superfamilies of transcription factors, play crucial roles in various functions, especially developmental processes. Thus, it is important to conduct a comprehensive identification and characterization of the NAC transcription factor family at the genomic level in *M. acuminata*. In this article, a total of 181 banana NAC genes were identified. Phylogenetic analysis indicated that NAC genes in *M. acuminata*, Arabidopsis, and rice were clustered into 18 groups (S1–S18), and MCScanX analysis disclosed that the evolution of MaNAC genes was promoted by segmental duplication events. Expression patterns of NAC genes during banana fruit ripening induced by ethylene were investigated using RNA-Seq data, and 10 MaNAC genes were identified as related to fruit ripening. A subcellular localization assay of selected MaNACs revealed that they were all localized to the nucleus. These results lay a good foundation for the investigation of NAC genes in banana toward the biological functions and evolution.

## 1. Introduction

Since many metabolic pathways in plants are regulated at the transcriptional level, transcription factors (TFs) play an important role in functional genomics [1]. After the discovery of the first transcription factor in maize approximately two decades ago [2], numerous TFs involved in various physiological processes and regulatory networks in higher plants have since been elucidated [1]. The function of TFs toward structural genes can be activated or repressed [3], and the activity of the transcription factor itself can be modulated by different signals resulting in controlled responses [4]. Therefore, it is important to study transcription factor families during postgenomic research.

Among numerous TFs, NAC (NAM, ATAF, CUC2) proteins constitute one of the largest plant-specific transcription factor gene families [5], with 105 in Arabidopsis [6], 140 in rice [7], 152 in soybean [8], 163 in *Populus trichocarpa* [9], and 180 in apple [10]. The N-terminus of the NAC domain is conserved while the C-terminus is highly divergent, which is responsible for the transcriptional activation involved in various processes such as developmental programs [11,12,13,14,15], defense [16,17], and responses to abiotic stress [17,18,19,20], among others. NAC TFs have been shown to exhibit important effects in ethylene biosynthesis, reception, and signaling during the fruit ripening of tomato [12]. Nieuwenhuizen et al. described the binding of three NAC TFs (AaNAC2, AaNAC3, and AaNAC4) to the promoter of AaTPS1, which is a terpene synthase gene in ripe *Actinidia arguta* fruit [21]. RIM1, a NAC TF found in rice, is degraded in response to jasmonate treatment and exhibits a root growth inhibition phenotype [19]. The expression profiles of NAC genes in apple indicated that MdNAC1a and MdNAC78 were repressed by ethylene and induced by 1-MCP during storage; another eight MdNACs were upregulated by ethylene, and their transcription reflected ethylene production rates during storage [22]. In brief, NAC transcription factors are abundant in plants with distinct structures and exert vital functions, especially in plant development [23].

Banana (*Musa acuminata*, AAA group) is an important fruit and food crop worldwide [24]. Additionally, its commercial value is largely dependent on the quality of the fruits, especially the shelf-life determined by the post-ripening of banana fruits. Recent studies have shown that many transcription factors play important roles in the regulation of banana fruit ripening. For instance, MaLBD1/2/3 can directly bind to the promoters of ripening-related MaEXP1/2 to regulate fruit ripening [25], and MaBSD1 can activate the expression of MaEXP1/2 to accelerate fruit ripening [26]. MaERFs can regulate banana fruit ripening directly in the transcriptional level or indirectly through interacting with ethylene biosynthesis genes [27]. Antisense or RNA interference (RNAi) of MaMADS1 and MaMADS2 hinders the color development and delays the softening of banana fruit, which is associated with the postponement of climacteric respiration and inhibited synthesis of ethylene [28]. Recently, an increasing number of NAC proteins have been functionally characterized in banana. Six NAC genes isolated from banana (*Musa acuminata* AAA group, cv. Cavendish), named MaNAC1-MaNAC6, were investigated, which suggested that MaNACs might be involved in banana fruit ripening via interactions with ethylene signaling components [20]. MaNAC1 promotes the ripening of banana and is regulated by ethylene and propylene, the latter of which induces the cold tolerance of banana fruits [29]. In addition, *MaNAC1*, *MaNAC2*, and *MaNAC5* are upregulated after infection by *Colletotrichum musae* and significantly enhanced by treatment with salicylic acid and methyl jasmonate [30]. A stress-associated NAC transcription factor, MpSNAC67, from *Musa x paradisiaca* has been shown to regulate senescence by promoting chlorophyll-catabolic genes [31]. A few vascular-related NAC transcription factors (MusaVND1, MusaVND2, and MusaVND3) have been reported to be efficient regulators of multiple classes of secondary wall-associated genes in banana [32]. The stomatal closure could be induced by a banana NAC protein, MusaSNAC1, by elevating H_2_O_2_ content in guard cells during drought stress [32]. Even though many NAC TFs in banana have been identified with respect to their function in fruit development and responses to biotic and abiotic stress, global genomic analysis of NAC genes in banana is still limited to NAC orthologous group analysis [33]. Therefore, the genome-wide identification and characterization of NAC TFs in banana could provide some hints to researchers interested in studying the NAC gene. Moreover, bananas, regarded as important food crops in both tropical and subtropical regions [34], are typical ethylene-depended climacteric fruits. Considering the crucial role of NAC TFs during banana ripening, it is necessary to conduct whole-genome identification of NAC genes to better understand the ripening metabolic pathways in banana.

In the present study, the total NAC genes in *M. acuminata* (designated as MaNACs) were identified according to the information supplied by the released ‘DH-Pahang’ genome in 2012 [24], and a phylogenetic analysis of MaNACs was conducted with comparisons to Arabidopsis and rice. A comprehensive investigation of the gene structure, motif composition, chromosome localization, gene duplication, and interchromosomal relationships was performed for MaNACs. Expression patterns of MaNACs during banana fruit ripening induced by ethylene at different time intervals were also examined. Then, 10 highly upregulated MaNACs were selected as candidate ethylene-responsive TFs. The mRNA expression levels of these MaNACs were quantified by quantitative real-time polymerase chain reaction (qRT-PCR), and their subcellular localization was also examined.

## 2. Results and Discussion

### 2.1. Identification of NAC Genes in M. Acuminata

A hidden Markov model (HMM) profile of the Pfam NAC domain (PF02365) was employed as a query to identify the NAC genes in *M. acuminata* (release 2.0, https://banana-genome-hub.southgreen.fr/). Initially, a total of 185 sequences were obtained. Subsequently, four sequences were removed due to the absence of the NAM domain or incompleteness of the genome assembly. Finally, 181 NAC genes in *M. acuminata* were identified and confirmed. Although some banana NAC genes have been published and named [29,30], we designated all the NAC genes as MaNACXXX, according to the nomenclature proposed in previous studies [6,9]. The reported NAC genes in *M. acuminate* are summarized in Table 1 with the gene name and ID used in the present study.

The information for these MaNACs is presented in Table S1 including the annotation number and description in the banana genome database, amino acids (AAs), molecular weight (Mw), and isoelectric point (pI). The identified MaNAC genes encoded proteins ranging from 119 (MaNAC015) to 1031 (MaNAC074) AAs in length, with an average of 333 AAs. The 181 MaNACs had a Mw ranging from 13.45 (MaNAC015) to 114.29 (MaNAC074) kDa, with an average of 37.35 kDa. In terms of pI, the values ranged from 4.24 (MaNAC145) to 11.00 (MaNAC169). The NAC gene family in *M. acuminata* is large compared with those identified in Chinese cabbage (204) [38], apple (180) [10], *Populus trichocarpa* (163) [9], *Gossypium raimondii* Ulbr. (145) [39], *Oryza sativa* (140) [7], potato (110) [40], *Arabidopsis thaliana* (105) [6], *Glycine max* (101) [41], Cassava (96) [42], and *Vitis vinifera* (79) [43].

### 2.2. Chromosomal Localization and Duplication Analysis of MaNAC Genes

**Figure 1:**
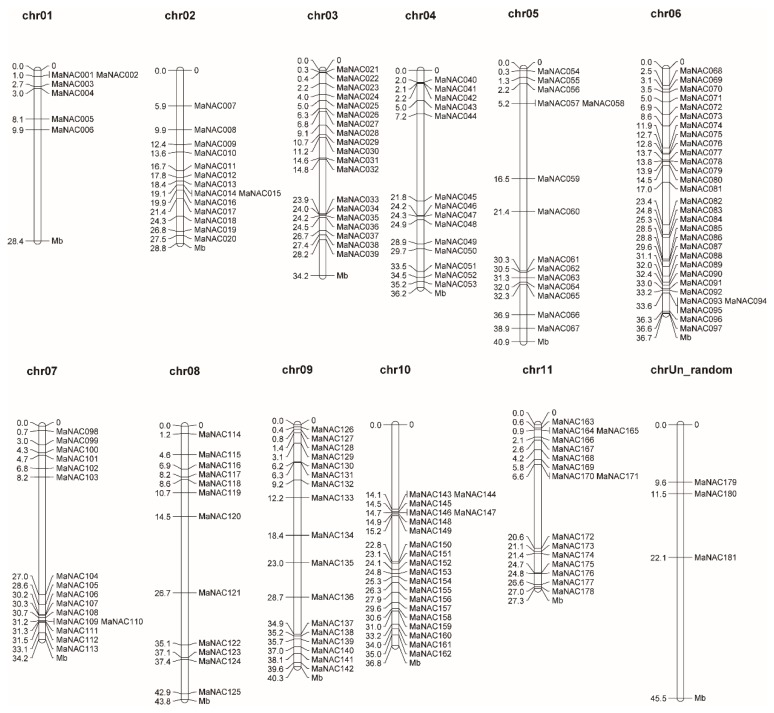
Chromosomal locations of NAC genes in *M. acuminata*. The chromosomal position of each MaNAC was mapped according to the genome of *M. acuminata*. The chromosome number is marked at the top of each chromosome and the unit for the scale is megabases (Mb).

**Figure 2:**
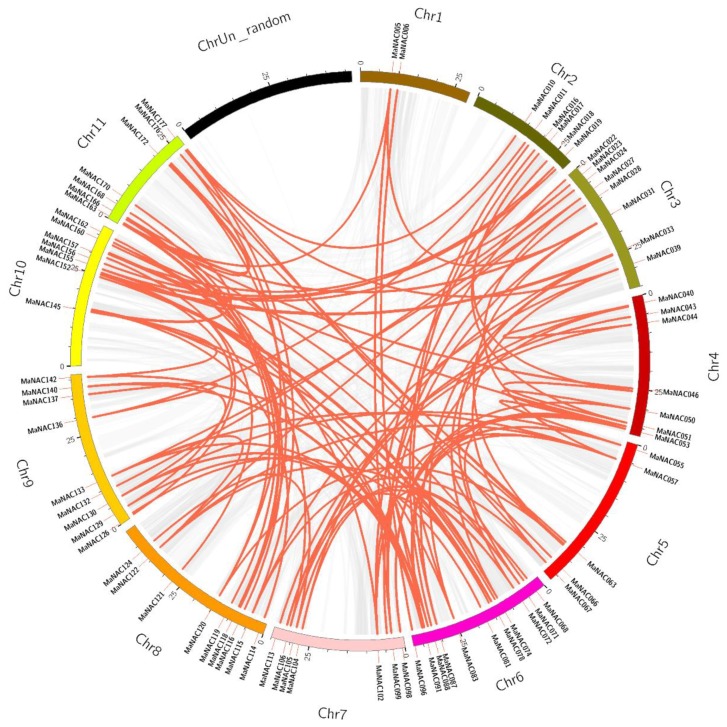
Schematic representation of the interchromosomal relationships of NAC genes in *M. acuminata*. Gray lines indicate all syntenic blocks in the banana genome, whereas orange lines suggest duplicated NAC gene pairs. The chromosome number is indicated at the top of each chromosome (color figure online).

All the MaNACs genes were mapped on the chromosomes, according to their positions given in the banana genome database using MapChart software. As illustrated in Figure 1, 181 MaNAC genes were unevenly distributed across all 12 chromosomes. Chromosome 6 (chr06) included the largest number (30) of MaNAC genes, followed by 20 on chr10. In contrast, only three genes were found on chrUn_random. The large number of MaNACs might due to gene duplication events as described previously in the *Musa* lineage, in which three rounds of whole-genome duplication (WGD) occurred [24]. In addition to WGD, tandem duplication and segmental duplication also play a vital role in the expansion of the large gene families in plants [44]. Therefore, BLASTP and Multiple Collinearity Scan Toolkit (MCScanX) were employed to analyze the duplication events for each MaNAC gene with default parameters [45], and the results were visualized by Circos and are illustrated in Figure 2. According to the analysis, four genes were clustered into two tandem duplication events (MaNAC001/002 and MaNAC014/015) (Figure 1). Additionally, 17 segmental duplication events with 30 MaNAC genes (detailed information provided in Table S2) were also identified. Furthermore, some MaNAC genes were involved in two or three duplication events such as MaNAC041 with MaNAC044 and MaNAC107, MaNAC107 with MaNAC041 and MaNAC162, and MaNAC091 with MaNAC089, MaNAC131, and MaNAC140. These results suggest that some of the NAC genes in banana might be produced by gene duplication, and undoubtedly the evolution of MaNAC genes is promoted by segmental duplication events. As illustrated in Figure 2, MaNAC genes were located within synteny blocks on almost all chromosomes except chrUn_random, which contained the least number of MaNACs. The collinearity assay demonstrated that at the whole-genome level, there were 16,160 colinear genes localized on 879 syntenic blocks covering 45.81% of the total genes in banana. In addition, 123 MaNACs (marked in red in Table S1) were colinear genes localized on 117 syntenic blocks, which covered 67.96% of the total MaNAC genes. The detailed 117 collinear pairs are shown in Table S3. To better understand the evolutionary constraints of the MaNAC gene family, Ka/Ks ratios, and divergence time of MaNAC, gene pairs were calculated and estimated as described previously [24,46]. As presented in Table S4, the Ka/Ks of all MaNAC gene pairs were less than one, indicating that the MaNAC gene family might have been subject to purifying selection and functional constraints during its evolution, which is consistent with the NAC genes reported in *Populus trichocarpa* [47].

### 2.3. Phylogenetic Analysis of NAC Genes in M. Acuminata, Arabidopsis and Rice

**Figure 3:**
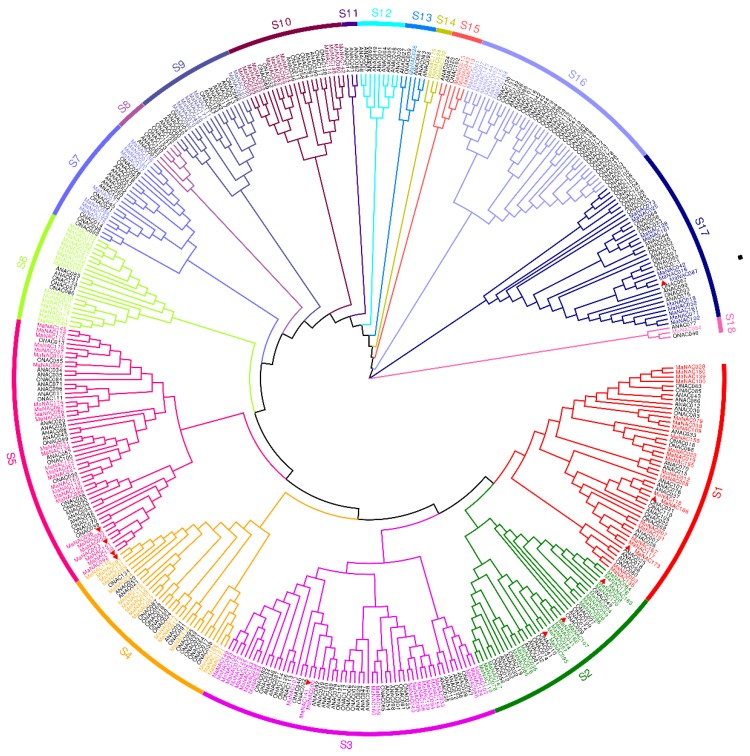
Phylogenetic analysis of NAC transcription factors from Arabidopsis, rice, and banana. ClustalW was applied for the alignment of protein sequences. Neighbor-joining method with 1000 bootstrap replicates was utilized to construct the phylogenetic tree in MEG7.0 software. Each NAC subfamily is indicated in a specific color. NAC proteins from banana are denoted in red.

To investigate the evolutionary characteristics of the NAC gene family, comparative phylogenetic and subgroup analyses of NAC protein sequences from banana, Arabidopsis, and rice were conducted. Figure 3 clearly shows that the phylogenetic tree divided the NAC family proteins into 18 distinct subgroups, consistent with the classification of NAC from *Populus trichocarpa* [9], and were numbered S1–S18 successively. Remarkably, none of the MaNAC genes was distributed in the S8, S11, and S12 subgroups, and ANAC065, ONAC005, ONAC015, ONAC048, and ONAC059 were excluded from the phylogenetic analysis since they formed individual subgroups without any MaNACs and were also located in the highest clades of the tree. This finding suggests the loss of these corresponding members during the divergence of *M. acuminata*. Interestingly, in addition to the clades containing only Arabidopsis, all the other subgroups included NAC genes from both monocots and dicots, which does not coincide with the results reported by Le et al. [8], who claimed that NACs from monocots and dicots are evolutionarily distinct.

The largest number of MaNAC genes was observed in the S5 subgroup with 27 MaNACs, followed by the S3 (24) and S1 (22) subgroups. However, the subgroup of S14 contained only three NAC genes (MaNAC024, MaNAC132, and MaNAC175) from banana and none from either Arabidopsis or rice, indicating that the three NAC genes might have been acquired in *M. acuminata* after divergence or loss in Arabidopsis or rice [9] as well as the specialized roles of these MaNACs [48]. Functional analysis of these genes might provide more information for the evolution of NAC transcription factors. In general, the number of MaNAC genes was predominant in the majority of the clades, especially in the S5 subgroup with the largest number of MaNACs.

Moreover, the S18 subgroup consisted of three NAC genes, each from *M. acuminata*, Arabidopsis, and rice, which suggested that these genes might have already been present before the divergence of the species [48]. The detailed relationship of orthologous NAC genes among *M. acuminata*, Arabidopsis, and rice also revealed that 40 orthologous groups of NAC sequences were probably derived from an ancestor gene [33]. Furthermore, the collinear pairs (as shown in Figure 2 and Table S3) were grouped closely together (e.g., MaNAC105 and MaNAC124 in S2; MaNAC122 and MaNAC133 in S3; MaNAC121 and MaNAC157 in S4; MaNAC098, MaNAC050 in S5, etc.), which suggests that the gene duplication occurred during its evolution. Consequently, expansion of the NAC genes in banana was mainly due to duplication events, which is consistent with the above result. In addition, gene structure and motif analysis of MaNAC genes were also conducted to further validate the subgroup classification. Notably, the reported NAC genes in *M. acuminata,* as presented in Table 1, are labeled with a red triangle in Figure 3. It can be seen that some of them grouped together and, thus, might exert similar functions. However, in the study conducted by Shan et al., MaNAC087 and MaNAC092 upregulated by ethylene were distributed in S17 and S5, individually as well as MaNAC098 (S5), MaNAC140 (S4), and MaNAC163 (S2) induced by ethylene [20]. These phenomena might be due to the complex roles played by MaNAC genes during fruit ripening. Moreover, some of these MaNAC genes might have diverse functions, for example, MaNAC140 also cooperates with MaWRKYs to enhance the expression of pathogenesis-related genes against *Colletotrichu musae* [30], and MaNAC087 is also involved in cold stress through interacting with MaCBF1 [29]. MaNAC genes are also capable of responding to abiotic stress. For instance, MaNAC095 in S2 can promote drought tolerance by modulating stomatal closure and H_2_O_2_ content [32], and MaNAC053 in S5 might be involved in responses to higher salinity and drought stresses in banana [37].

### 2.4. Gene Structure and Motif Analysis of MaNAC Genes

**Figure 4:**
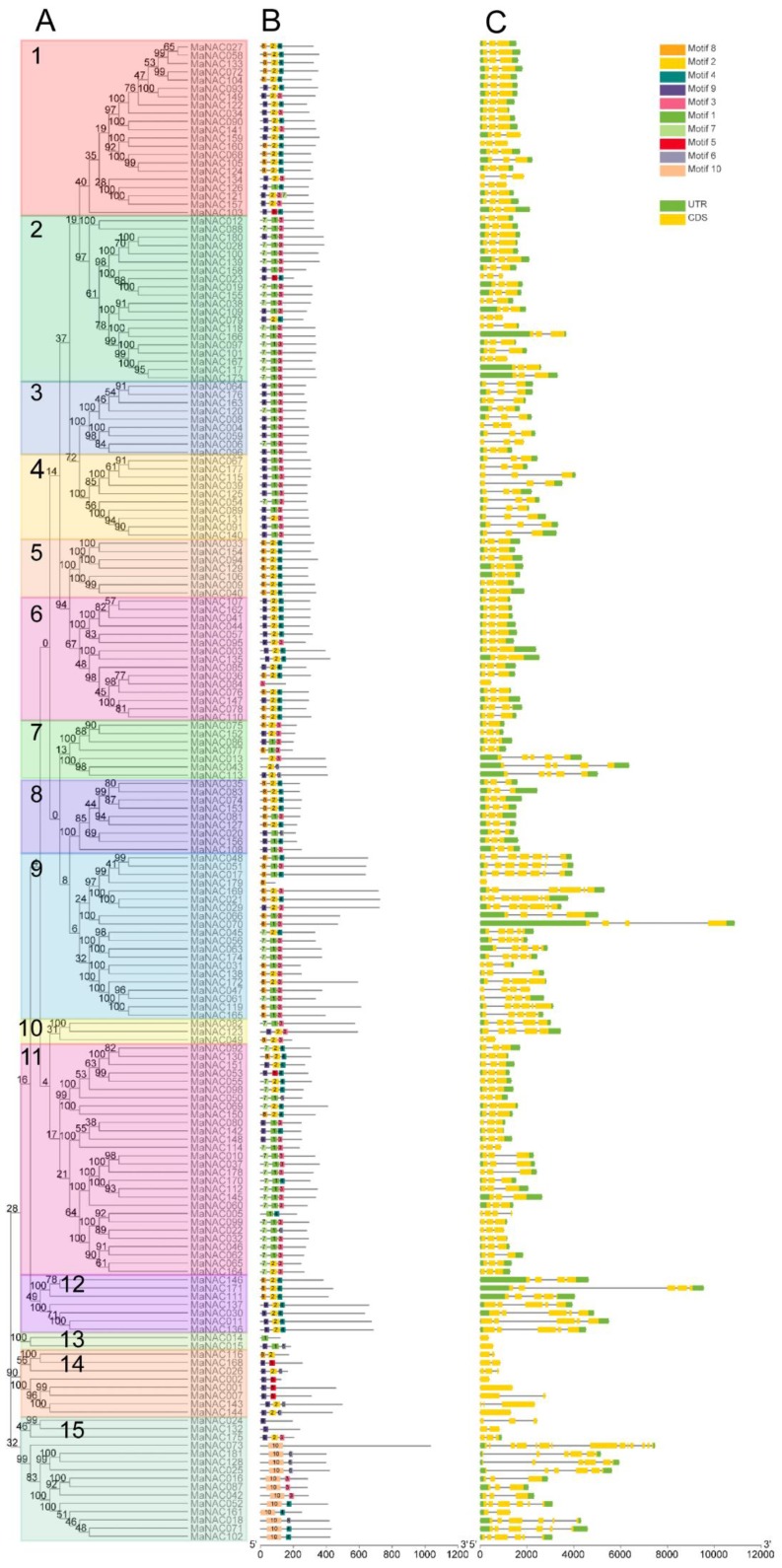
Phylogenetic relationships, motif compositions, and gene structure of banana NAC genes. (**A**) Multiple alignments of 181 full-length amino acids of NAC genes from *M. acuminata.* The evolutionary tree was constructed by the neighbor-joining method using 1000 bootstrap replicates, and the percentage of bootstrap scores was labeled at each node. Each subfamily was designated numerically and marked with individual color backgrounds. (**B**) Conserved motifs analysis of the MaNAC proteins. All motifs were identified by the MEME database with the complete amino acid sequences of MaNACs. The detailed information for each motif was provided in Figure S1. (**C**) Gene structure of banana NAC members. Exons and introns are represented by yellow boxes and black lines, respectively, the UTR (Un-Translated Region) is marked in green. The scale for the estimation of the sizes of exons and introns is presented at their bottom.

Based on the alignment of the proteins from 181 MaNACs, a phylogenetic tree (Figure 4A) was constructed using MEGA 7.0 by the neighbor-joining (NJ) method with 1000 bootstrap replicates. The motif architectures and gene structures of 181 MaNACs were annotated within the phylogenetic context and visualized by TBtools [49], as illustrated in Figure 4B,C. According to frequencies of occurrence, motif 1 and motif 2 were the two most frequently presented motifs, covering almost all the subgroups from S1 to S14. Motif 10 was a specific motif that was solely detected in S15. Subgroup S11 contained the largest number of motifs, from motifs 1 to 9. To gain more insight into the evolutionary relationships within the banana NAC gene family, the exon–intron organizations of all MaNAC genes were examined. As shown in Figure 4C, among the 181 MaNACs, 7 had no introns, 174 possessed two to 14 exons (6 with two exons, 129 with three exons, 8 with four exons, 16 with five exons, 9 with six exons, 5 with seven exons, and 1 with 14 exons). In addition, a phylogenetic tree of all 181 MaNAC genes divided them into 15 subgroups (Figure 4A). Genes within the same group usually had a similar and conserved structure in terms of exon number and intron length; for example, all the members of groups 1 to 5 contained three exons and two introns. Nevertheless, the gene structures in groups 9, 12, and 15 were variegated and distinct. All introns among the total 181 MaNAC genes were in phase 1, which suggested that they were highly conserved [9]. This result, which is consistent with the NAC genes reported in *Populus trichocarpa,* indicated that exon shuffling occurred during the evolutionary process [9,50]. The clustered MaNAC pairs presented conserved motifs and exon/intron distributions such as MaNAC181/128, MaNAC004/059, MaNAC019/155, and MaNAC041/107, among others. However, it is worth noting that in the pairs of MaNAC043/113, MaNAC081/127, and MaNAC091/131, the motif compositions were slightly different. Additionally, among the MaNAC146/171 and MaNAC171 pairs, one contained one more exon/intron than the other. This result indicated that during the structural evolution of MaNAC paralogs, single intron loss or gain occurred. Moreover, the conserved motifs and exon/intron structures of these paralogous pairs further verified the analyses of the phylogenetic tree and duplication events.

### 2.5. Characterization of Fruit-Ripening-Related MaNAC Genes

**Figure 5:**
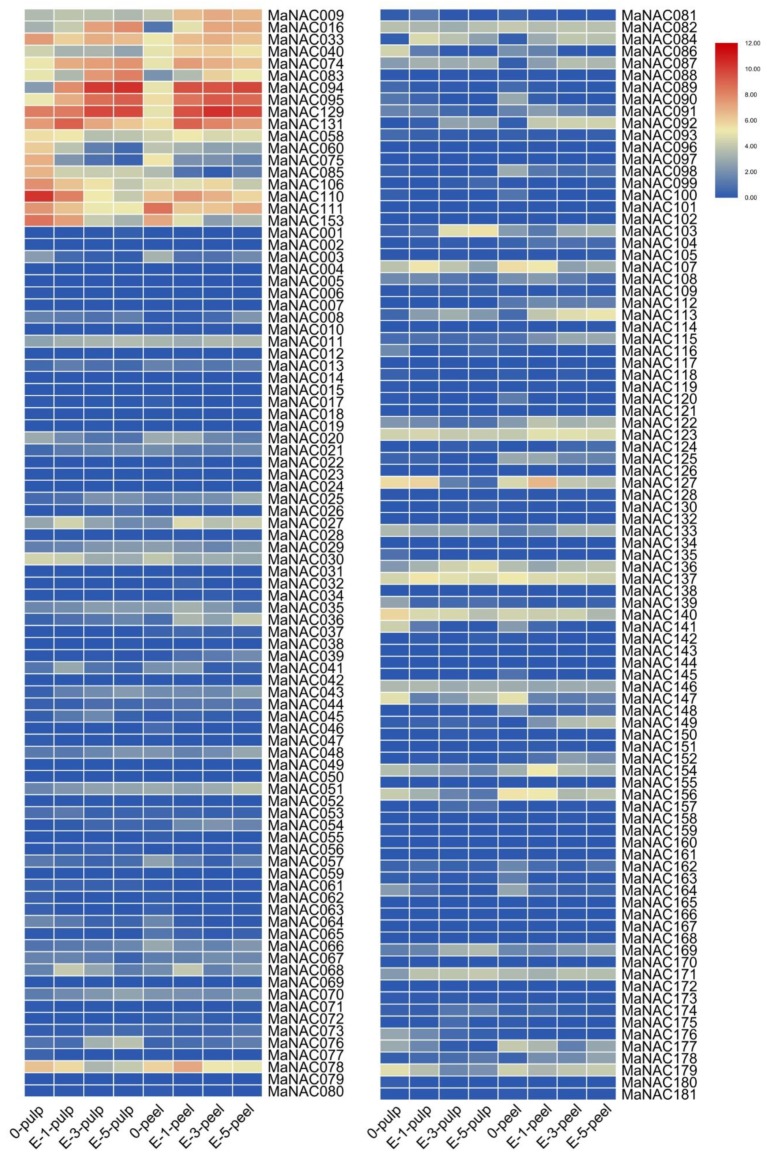
Expression patterns of MaNAC genes in the pulp or peel of banana at different intervals after treatment with ethylene (E represents ethylene, the number indicates the days after treatment). The heat map was generated using TBtools. The bar at the right of the heat map represents relative expression values.

To identify MaNAC genes related to fruit ripening, transcriptome data for banana fruit treated with exogenous ethylene were obtained from Illumina RNA-Seq data generated in this study. The heatmap was created based on fragments per kilobase of transcript per million fragments mapped (FPKM) values from different samples. As shown in Figure 5, in untreated fruit, most of the expression levels of MaNAC genes were relatively low, excluding nine genes (MaNAC033/075/085/106/110/111/129/131/153) with high expression levels in the pulp (FPKM > 100) and four genes (MaNAC078/110/111/153) with relatively high expression levels in the peel (FPKM > 50). Among them, three genes (MaNAC110/111/153) exhibited high expression levels in both the pulp and peel (FPKM > 80). Based on the data from ethylene-treated samples, we found that the expression levels of the 30 and 32 MaNAC genes were 2-fold upregulated in pulp and peel at day 5 after ethylene treatment, respectively (Table S5). However, when we evaluated these data carefully, some of the genes with high fold change values showed very low expression levels, suggesting that they might not be induced by ethylene. Subsequently, 10 MaNAC genes (MaNAC009/016/033/040/074/083/094/095/129/131) were selected as potentially related to the ripening of banana, according to their expression levels and fold change values after treatment with ethylene. All 10 MaNACs were upregulated in the fruits of banana induced by ethylene. Of them, only MaNAC094 exhibited extremely high expression in both the peel and pulp after ethylene treatment. MaNAC083 and MaNAC103 also demonstrated a similar expression pattern in pulp; however, MaNAC009 showed upregulated expression only in peel treated with ethylene. The results indicated that these 10 MaNAC genes might be involved in fruit ripening.

**Figure 6:**
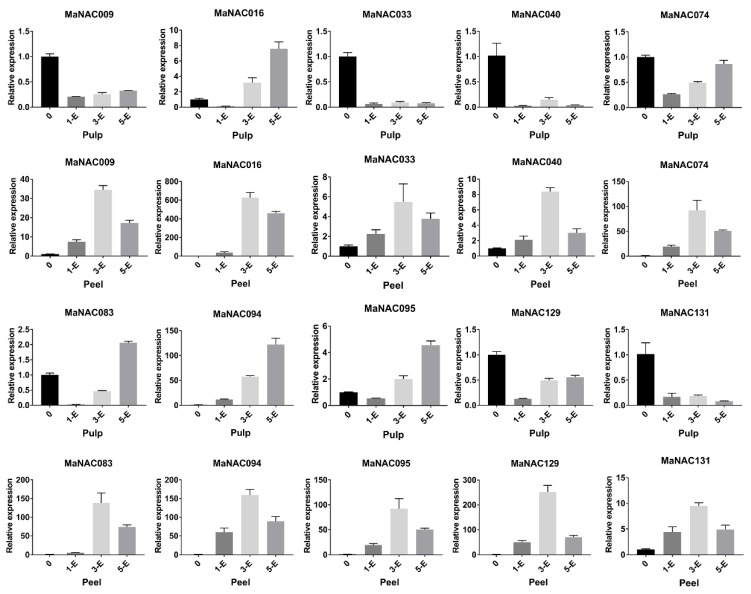
Relative expression of 10 selected MaNAC genes in the pulp or peel of banana at different intervals after treatment with ethylene (E represents ethylene, the number indicates the days after treatment). qRT-PCR data were normalized using the CAC gene. The name of the gene is indicated above each bar diagram. Error bars indicate the standard deviation.

Moreover, recent research has shown that MaNAC087 might be involved in fruit ripening and cold stress [20,29], and MaNAC094 is considered a critical regulator of the banana ethylene signal transduction pathway by omics analysis [51]. Further genetic evidence is required for a full understanding of their role in banana fruit ripening. To further verify the expression patterns of these 10 genes, qRT-PCR was carried out, and the data are presented in Figure 6. Expression of the 10 MaNAC genes revealed identical patterns based on the RNA-Seq and qRT-PCR data. Furthermore, the 10 MaNACs were all upregulated after treatment of the peel with ethylene, indicating that the concerned 10 MaNAC genes were directly related to the ripening of banana peel. Regarding their expression in the pulp, we can see that MaNAC016, MaNAC083, MaNAC094, and MaNAC095 were significantly upregulated by ethylene, which demonstrated that these genes function in the pulp-ripening process of banana.

### 2.6. Subcellular Localization Analysis of Selected MaNACs

**Figure 7:**
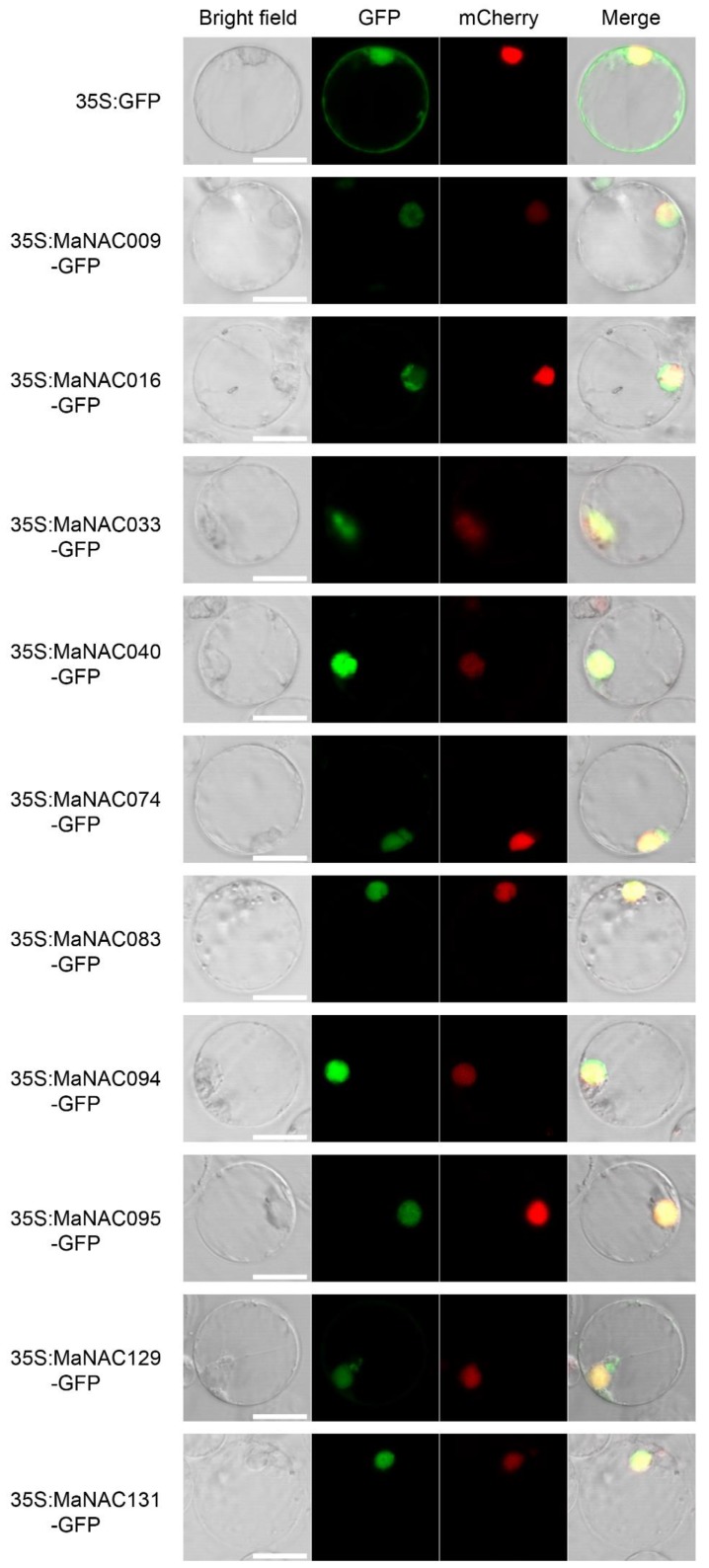
Subcellular localization of the selected 10 MaNACs cloned from *M. acuminata* and used to construct 35S:MaNAC-GFP vectors, in which GFP (Green Fluorescent Protein) was fused at the C-terminus. The fusion proteins, as well as GFP as the control, were transiently expressed in rice protoplasts and observed by fluorescence microscopy. The merged images include the green fluorescence channel (second panel) and the nuclear mCherry m9arker of the e-channel (third panel). The corresponding bright field images are shown in the first panel. Bar = 5 μm.

Protein subcellular localization is important to elucidate protein function. To determine the subcellular localization of these selected 10 MaNAC genes, 35S:GFP- MaNAC009/016/033/040/074/083/094/095/129/131 and 35S:GFP were transiently expressed in rice protoplasts. As shown in Figure 7, the rice cells transformed with p35S:GFP vector displayed fluorescence throughout the whole cell. In contrast, all the fluorescence in cells transformed with p35S:GFP-MaNAC009/016/033/040/074/083/094/095/129/131 was detected exclusively in the nucleus, suggesting that all 10 selected MaNAC genes encoded nuclear proteins, consistent with their putative role in transcriptional regulation as transcription factors.

### 2.7. Cis-Element Analysis of Selected MaNACs

**Figure 8:**
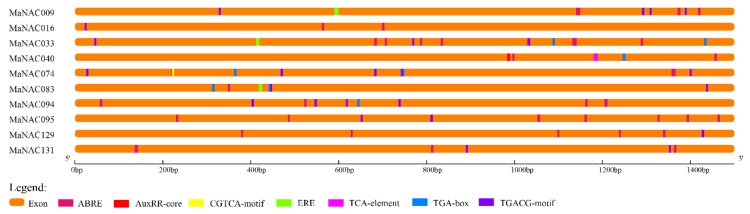
*Cis*-element analysis of 10 selected MaNAC genes from the upstream 1500 bp sequence to the transcription start site.

In Figure 8, the *cis*-elements of the 10 MaNAC genes are illustrated to further examine the regulatory mechanisms of MaNACs related to fruit ripening. Ethylene-responsive elements (ERE, ATTTCAAA) were discovered in three genes (MaNAC009/033/083) involved in fruit ripening [52], but they were not detected in the other seven MaNAC genes. This result indicates that these genes containing ERE might play a direct role in response to ethylene signaling, and the other five genes are involved in fruit ripening in complex ways. Notably, all the selected MaNAC genes contained the TGACG-motif and CGTCA-motif, which are *cis*-acting regulatory elements involved in MeJA (jasmonic acid methyl ester) responsiveness, excluding MaNAC040. MeJA has been reported to be involved in fruit ripening; for instance, it activates MYC2 to regulate MdERs and ethylene biosynthetic genes in apple [53]. All 10 MaNACs included the abscisic acid-responsive element (ABRE, GACACGTGGC), which is related to fruit ripening in an ABA-dependent manner in strawberry [54]. In addition, MaNAC040, MaNAC074, MaNAC083, and MaNAC094 contained the TGA-box (AACGAC), which is an auxin-responsive element, and MaNAC040 included the AuxRR-core (GGTCCAT), which is also a cis-acting regulatory element involved in auxin responsiveness. A previous study has reported that ethylene and auxin can control tomato fruit metabolism by participating in light signaling cascades [54]. Consequently, these genes make important contributions to the expression of the corresponding genes during fruit ripening. Further investigation of these 10 MaNAC genes might provide important information regarding the molecular mechanism of fruit ripening.

## 3. Methods

### 3.1. Identification of NAC Genes in M. Acuminata

A hidden Markov model (HMM) profile of the NAC domain downloaded from the Pfam protein family database (available online: http://pfam.sanger.ac.uk/) was adopted for the identification of NAC genes (NACs) in the *Musa acuminata* genome (available online: https://banana-genome-hub.southgreen.fr/) using HMMER3.2.1. Default parameters were employed, and the cutoff value was set to 0.01. To confirm the presence of the NAC domain, a batch search of the sequences of all the obtained MaNAC genes was conducted using the online databases SMART (available online: http://smart.embl.de/smart/set_mode.cgi?GENOMIC=1), (The National Center for Biotechnology Information Conserved Domain Database) (available online: https://www.ncbi.nlm.nih.gov/cdd/) and PFAM (available online: http://pfam.xfam.org/). Redundant sequences were manually deleted. Characteristics such as the amino acid number, molecular weight, and isoelectric point of the identified MaNAC genes were obtained from the ExPasy website (available online: http://web.expasy.org/protparam/).

### 3.2. Phylogenetic Analysis of NAC Genes in M. Acuminata, Arabidopsis, and Rice

The full-length protein sequences of MaNACs, ANACs, and ONACs were aligned by ClustalW in Mega7 with 1000 bootstrap replicates. Then, to ensure that the topology of the NJ tree contained more divergent C-terminal domains, the pairwise gap deletion mode was applied for the construction of the unrooted phylogenetic tree [9].

### 3.3. Gene Structure and Motif Analysis of MaNAC Genes

An online program of the gene structure display server (GSDS2.0) (available online: http://gsds.cbi.pku.edu.cn/index.php) was applied to draw the exon/intron organization of each MaNAC gene by comparing the cDNAs with their corresponding full-length sequences [55]. The MEME tool (version 5.0.4, Washington, DC, USA, http://meme-suite.org/tools/meme) was used to identify conserved motifs of these MaNACs proteins [56]. Parameters applied were as follows: the number of motifs searched was set as 10, and the limits of motif widths were between six and 50 residues.

### 3.4. Chromosomal Localization and Duplication Analysis of MaNAC Genes

All the MaNACs genes were mapped to the banana chromosomes with MapChart software, according to their positions in the database. The Multiple Collinearity Scan Toolkit (MCScanX, Athens, Greece) was employed to analyze the duplication events for each MaNAC gene using the default parameters [45].

### 3.5. Plant Material and Treatments

Pre-climacteric banana (*Musa acuminata*, AAA group, cv. Cavendish) fruits at the 75%–80% plump stage were obtained from the banana plantation at the Institute of Fruit Tree Research, Guangdong Academy of Agricultural Science. The hands were split into separate fingers, and fruits of uniform weight, shape, and maturity as well as free from visual defects were selected. First, the fruits were surface sterilized by dipping them into sodium hypochlorite solution (1%) for 1 min and immersed into a solution containing preservative (GENGREEN, Zhuhai, China) for 5 min to avoid fungal diseases. They were then dried at 25 °C for 2 h before treatment. The selected fruits were randomly divided into three biologically repeated groups, each of which was treated with ethylene (100 μL/L) for 18 h and then stored at 22 °C. Samples were fetched at time intervals of 0, 1, 3, and 5 days for the three groups. The pulps and peels of all samples were quickly separated and immersed in liquid nitrogen and then stored at −80 °C until utilization.

### 3.6. RNA Isolation and RT-PCR Analysis

The samples were ground into fine powder with liquid nitrogen, and extraction of total RNA was conducted according to a previously reported method [57]. The RNA was treated with gDNA Eraser (TaKaRa, Dalian, Japan) to eliminate any potential contamination with DNA. Then, DNA-free total RNA was reverse transcribed into cDNA using a reverse transcription kit (TaKaRa, Dalian, Japan) based on the manufacturer’s protocol. RT-PCR (Quantitative Real-time Polymerase Chain Reaction) was then performed using the Applied Biosystems StepOnePlus Real-Time PCR System (ThermoFisher, Woodlands, Singapore) using the following conditions: an initial denaturation step at 94 °C for 30 s, followed by 40 cycles of 30 s at 94 °C, and 5 s at 60 °C. The reaction was prepared in a 20-µL system including 8 µL template, 10 µL TB Green^®^ Premix (Tli RNaseH Plus, TAKARA, Dalian, China), 0.4 µL ROX, and 0.8 µL of each primer. The primers used in this experiment are listed in Table S6. CAC (clathrin adaptor complex) was used as a reference gene [58]. Gene specific primers for qRT-PCR analysis was designed with Beacon Designer 7 software, and primers producing a single product of correct size, and with 90–110% PCR amplification efficiency were used for further PCR assay. The expression levels were normalized using the cycle threshold (Ct) corresponding to that of the reference gene. Calculation of the relative expression level of the gene of interest was conducted as described previously [59], and the data are shown as the mean ± standard deviation of three replicates.

### 3.7. Subcellular Localization

The coding sequences of MaNACs without the stop codon were amplified by PCR (primers are listed in Table S7) and subcloned into the pCambia1300-GFP vector (modified from pCambia1300by in frame with the green fluorescent protein (GFP) sequence. The obtained 35S::gene–GFP cassette was derived by the cauliflower mosaic virus (CaMV) 35S promoter. The polyethylene glycol (PEG)-mediated transient expression assays were performed using 35S::gene–GFP vector and pCambia1300-GFP empty vector with rice protoplasts as described previously [60]. GFP fluorescence was observed using a fluorescence microscope (Zeiss LSM 710, Jena, Germany). All transient expression assays were repeated at least three times.

### 3.8. RNA-Sequencing Analysis

Banana fruits at the 75–80% plump stage was treated with ethylene, and fruit samples at different time points were collected for total RNA isolation using above-mentioned methods. RNA sequencing was conducted using an Illumina’s HiSeq 2500 sequencer producing 150 bp paired-end reads. The clean reads were analyzed with Tophat [61] (available online: http://tophat.cbcb.umd.edu/) for alignment to *M. acuminata* DH Pahang v2 (*M. acuminata*, A-genome, 2*n* = 22) reference genome [24], Cufflinks (available online: http://cufflinks.cbcb.umd.edu/) [62], and Cuffdiff (a package from Cufflinks) were used for transcriptome assemble and normalized expression level/differential expression calculation with multiple test correction, respectively. The pipeline was referred to Trapnell et al., 2012 [63].

### 3.9. Heat Map Construction and Cis-Element Analysis

The MaNACs gene expression determined by RNA-Seq were presented with heat map using TBtools [49]. The data was standardized by log-transformation using 2 as the base of the logarithm, and all the values of gene expression were plus 1 to avoid that some genes without expression are unable to be calculated. For cis-acting element analysis, genomic DNA sequences in the promoter region (−1500 to −1 bp) were scanned in the Plant CARE database (available online: http://bioinformatics.psb.ugent.be/webtools/plantcare/html/) [64].

## 4. Conclusions

In summary, 181 NAC genes in the banana genome were identified in this study. Based on a careful characterization of their chromosome location, duplication events, phylogenetic relationship with Arabidopsis and rice, structure and motif, the global analysis of this gene family in banana has been achieved. Moreover, with the help of RNA-Seq, 10 NAC genes potentially related to fruit ripening were selected and characterized by qRT-PCR, *cis*-element analysis, and subcellular localization observation. The results indicate that 4 MaNACs and 10 MaNACs might be closely involved in the ripening of the banana pulp and peel, respectively, and in the promoter of 3 MaNAC genes ethylene-responsive elements were discovered. In addition, all the 10 MaNACs were localized in the nuclear zone. These studies and analyses provided an overview information regarding the NAC genes in *M. acuminata* and determined the candidate NAC genes that regulating banana ripening for further investigation.

## Figures and Tables

**Table 1:** Reported NAC genes in M. *acuminata* in the literature.

Gene Name in the Literature	Gene ID	Annotation Number	Functions Involved	References
MaNAC1	MaNAC087	Ma0628730.1	Ethylene signaling; cold stress	[20,29]
MaNAC2	MaNAC092	Ma0633280.1	Ethylene signaling	[20]
MaNAC3	MaNAC141	Ma0928160.1	Ethylene signaling	[20]
MaNAC4	MaNAC098	Ma0700860.1	Ethylene signaling	[20]
MaNAC5	MaNAC140	Ma0926680.1	Ethylene signaling	[20]
MaNAC6	MaNAC163	Ma1100790.1	Ethylene signaling	[20]
MusaVND2	MaNAC166	Ma1102940.1	Secondary wall deposition	[35]
MusaVND3	MaNAC173	Ma1116010.1	Secondary wall deposition	[35]
MusaNAC68	MaNAC107	Ma0723280.1	Auxin signaling	[36]
MusaNAC042	MaNAC053	Ma0438520.1	Drought and salinity tolerance	[37]
MusaSNAC1	MaNAC095	Ma0633990.1	Drought tolerance	[32]

## Supplementary Materials

Supplementary materials can be found at https://www.mdpi.com/1422-0067/21/2/634/s1.

## Abbreviations

MaNAC	NAC genes in *Musa acuminata*
ANAC	NAC genes in *Arabidopsis thaliana*
ONAC	NAC genes in *Oryza sativa*
HMM	Hidden Markov Model
WGD	Whole-Genome Duplications
MCScanX	Multiple Collinearity Scan toolkit
AAs	Amino Acids
Mw	Molecular weights
pI	Isoelectric points
GFP	Green Fluorescent Protein
