# Supplementary material for: Genome-Wide Identification and Characterization of the NAC Transcription Factor Family in Musa Acuminata and Expression Analysis during Fruit Ripening
Source: Int J Mol Sci. 2020 Jan 18;21(2):634. doi: 10.3390/ijms21020634 (PMC7013864; doi:10.3390/ijms21020634)
Supplement: Supplementary file 1 [file ijms-21-00634-s001.pdf]

Article

Genome-wide identification and characterization of the NAC transcription factor family in *Musa acuminata* and expression analysis during fruit ripening

Bin Li [1,2,3], Ruiyi Fan [2,3], Qiaosong Yang [2,3], Chunhua Hu [2,3], Ou Sheng [2,3], Guiming Deng [2,3], Tao Dong [2,3], Chunyu Li [2,3], Xinxiang Peng [1], Fangcheng Bi [2,3,*] and Ganjun Yi [2,3,*]

1 State Key Laboratory for Conservation and Utilization of Subtropical Agro-bioresources, College of Life Sciences, South China Agricultural University, Guangzhou 510642, China; binxiuke2343@126.com (B.L.); xpeng@scau.edu.cn (X.P.)

2 Key Laboratory of South Subtropical Fruit Biology and Genetic Resource Utilization(MOA), Institute of Fruit Tree Research, Guangdong Academy of Agricultural Sciences; Guangzhou 510640, China

3 Guangdong Province Key Laboratory of Tropical and Subtropical Fruit Tree Research, Institute of Fruit Tree Research, Guangdong Academy of Agricultural Sciences; Guangzhou 510640, China; fanruiyi@outlook.com (R.F.); yangqiaosong@gdaas.cn (Q.Y.); huchunhua@gdaas.cn (C.H.); shengou@gdaas.cn (O.S.); dengguiming@gdaas.cn (G.D.); dongtao@gdaas.cn (T.D.); lichunyu@gdaas.cn (C.L.)

* Correspondence: bifangcheng@gdaas.cn (F.B.); yiganjun@vip.163.com (G.Y.)

Motif 1

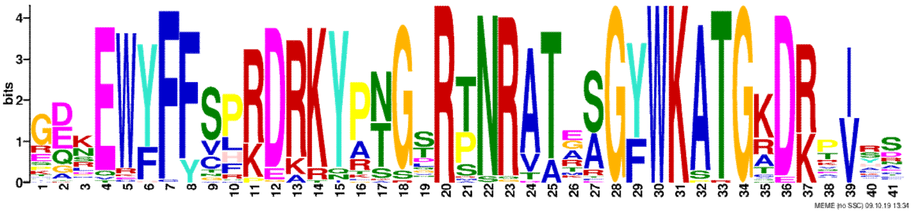

Motif 2

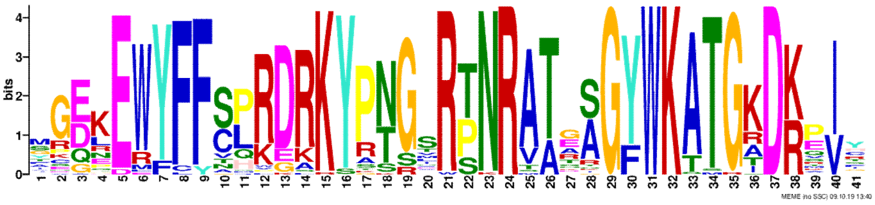

Motif 3

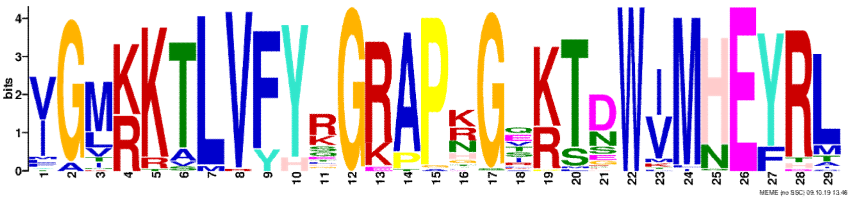

Motif 4

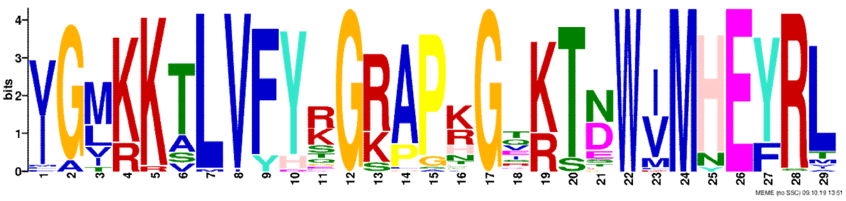

Motif 5

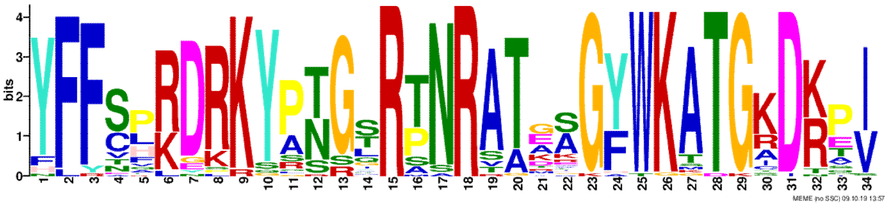

Motif 6

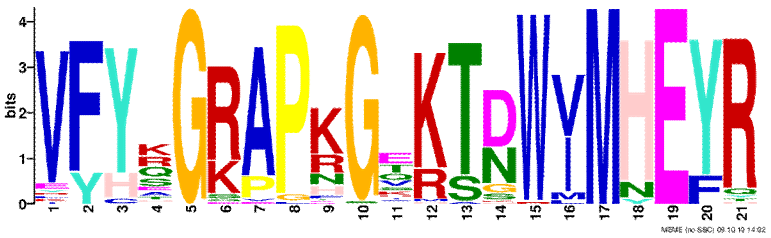

Motif 7

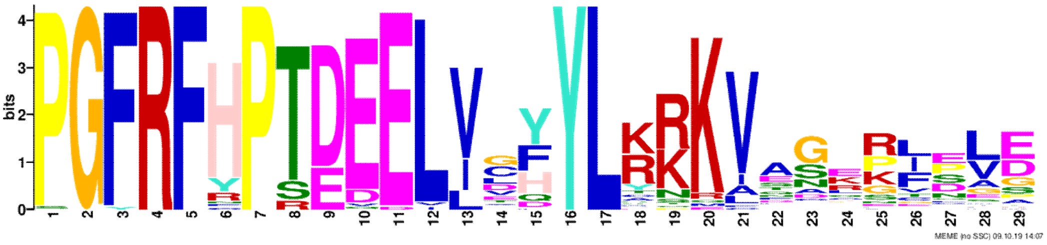

Motif 8

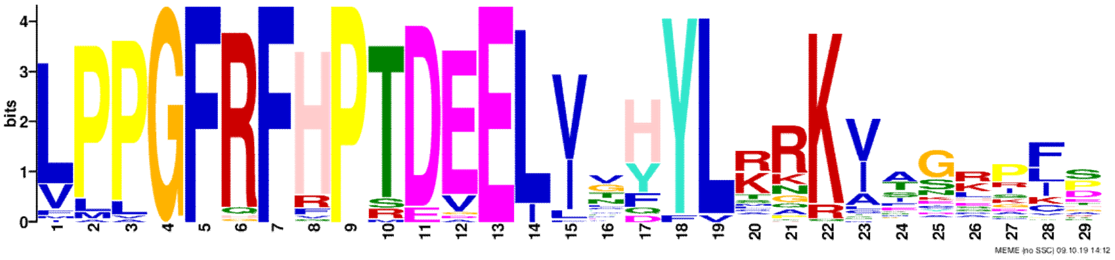

Motif 9

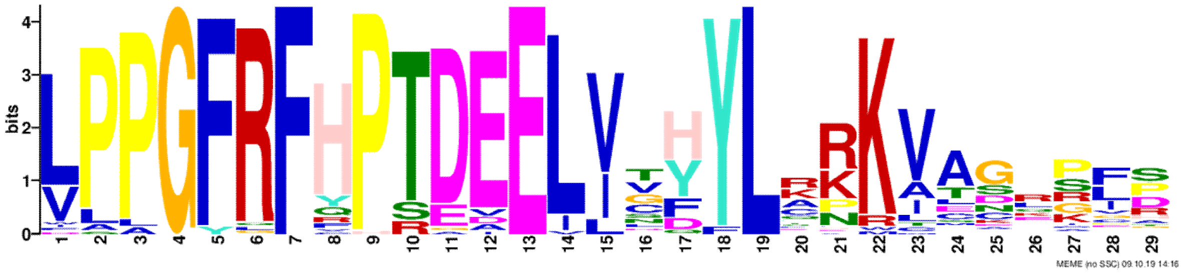

Motif 10

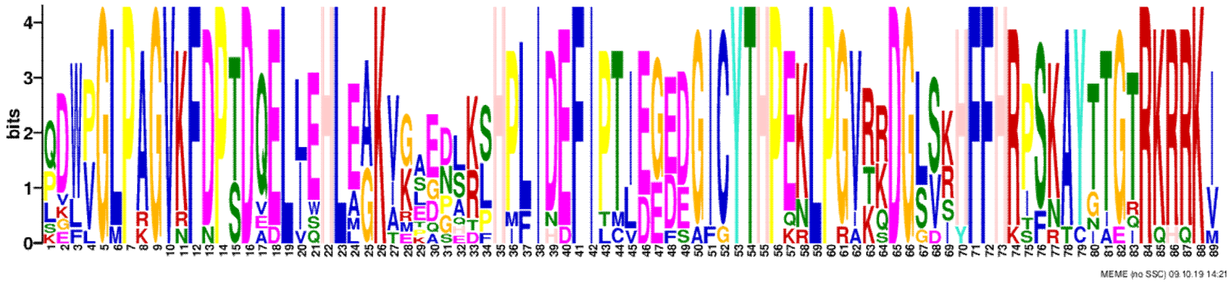

Figure S1. Sequence logos for the conserved motifs of banana NAC domain proteins. Conserved motifs and the sequence logos were generated using the MEME search tool. Numbers on the horizontal axis represent the sequence positions in the motifs and the vertical axis represents the information content measured in bits.

Table S1. Reported NAC genes from *Musa acuminate* in literatures.

Gene symbol	Annotation numbera	AAs	Mw	pI	Description
MaNAC001	Ma0101430.1	457	48763.5	4.28	Hypothetical protein
MaNAC002	Ma0101440.1	124	13487.2	9.43	Putative NAC domain-containing protein 41
MaNAC003	Ma0104220.1	392	42789.3	8.7	Putative NAC transcription factor NAM-2
MaNAC004	Ma0104590.1	292	33343.2	5.41	No apical meristem protein, putative, expressed
MaNAC005	Ma0111390.1	218	24632.3	8.88	Putative NAC domain-containing protein 68
MaNAC006	Ma0113870.1	278	31361.2	4.71	No apical meristem protein, putative, expressed
MaNAC007	Ma0200690.1	308	33180.9	8.33	Hypothetical protein
MaNAC008	Ma0201530.1	264	29553.7	5.23	No apical meristem protein, putative, expressed
MaNAC009	Ma0201890.1	328	36854.4	7.53	NAC transcription factor 29
MaNAC010	Ma0202630.1	328	37252.1	8.72	Uncharacterized protein
MaNAC011	Ma0206010.1	673	74415.3	4.63	No apical meristem protein, putative, expressed
MaNAC012	Ma0207640.1	325	36226.5	8.37	Protein BEARSKIN2
MaNAC013	Ma0208540.1	393	44116.4	4.42	No apical meristem protein, putative, expressed
MaNAC014	Ma0209680.1	119	13448.4	10.16	Putative NAC transcription factor 92
MaNAC015	Ma0209690.1	182	20625.6	10.01	Putative NAC transcription factor 29
MaNAC016	Ma0210970.1	286	32176.9	7.19	Uncharacterized protein
MaNAC017	Ma0213650.1	637	69809.4	5.08	Uncharacterized protein
MaNAC018	Ma0218350.1	417	47104.5	7.17	No apical meristem protein, putative, expressed
MaNAC019	Ma0221900.1	312	35563.1	7.56	Protein SOMBRERO
MaNAC020	Ma0223000.1	212	23683.8	9.26	NAC domain-containing protein 83
MaNAC021	Ma0300260.1	724	80835.6	4.53	No apical meristem protein, putative, expressed
MaNAC022	Ma0300480.1	280	32175.1	5.04	Putative Protein ATAF2
MaNAC023	Ma0303310.1	200	22982.9	8.23	NAC domain-containing protein 76
MaNAC024	Ma0305920.1	193	22206.2	4.33	Putative NAC domain-containing protein 94
MaNAC025	Ma0307280.1	417	46481.5	5.14	NAC domain-containing protein 8
MaNAC026	Ma0308760.1	163	18007.2	9.1	Putative Transcription factor JUNGBRUNNEN 1
MaNAC027	Ma0309370.1	319	36093.8	7.01	Putative NAC domain-containing protein 100
MaNAC028	Ma0311950.1	384	42253.7	7.24	NAC domain-containing protein 43
MaNAC029	Ma0313900.1	720	79555.7	4.52	No apical meristem protein, putative, expressed
MaNAC030	Ma0314290.1	632	69858.3	4.63	No apical meristem protein, putative, expressed
MaNAC031	Ma0315190.1	241	27472.7	4.7	NAC domain-containing protein 45
MaNAC032	Ma0315340.1	290	33350.5	4.89	No apical meristem protein, putative, expressed
MaNAC033	Ma0319100.1	323	36416.5	8.45	No apical meristem protein, putative, expressed
MaNAC034	Ma0319110.1	294	33195.3	8.09	No apical meristem protein, putative, expressed
MaNAC035	Ma0319490.1	236	26412.5	8.67	No apical meristem protein, putative, expressed
MaNAC036	Ma0319830.1	303	33525.5	7.96	NAC domain-containing protein 2
MaNAC037	Ma0322620.1	357	39982.9	8.03	No apical meristem protein, putative, expressed
MaNAC038	Ma0323470.1	303	34712.5	6.35	NAC domain-containing protein 76
MaNAC039	Ma0324610.1	281	31156.9	8.06	Putative NAC domain-containing protein 21/22
MaNAC040	Ma0402440.1	335	37313.6	8.33	No apical meristem protein, putative, expressed
MaNAC041	Ma0402450.1	300	34302.2	7.02	NAC domain-containing protein 68
MaNAC042	Ma0402750.1	289	31940	9.15	NAC domain-containing protein 73
MaNAC043	Ma0407120.1	398	43847.9	4.91	No apical meristem protein, putative, expressed
MaNAC044	Ma0410270.1	294	33448.6	7.96	NAC domain-containing protein 68
MaNAC045	Ma0419710.1	329	37608.2	5.33	No apical meristem protein, putative, expressed
MaNAC046	Ma0422510.1	273	31416	8.16	Putative NAC domain-containing protein 102
MaNAC047	Ma0422610.1	373	41752.4	5.31	No apical meristem protein, putative, expressed
MaNAC048	Ma0423340.1	651	72939.2	4.89	Uncharacterized protein
MaNAC049	Ma0428500.1	188	21728.2	8.98	Putative NAC transcription factor ONAC010
MaNAC050	Ma0429550.1	251	28758.1	8.04	Putative NAC domain-containing protein 68
MaNAC051	Ma0435660.1	640	71150.1	4.41	Uncharacterized protein
MaNAC052	Ma0437470.1	408	46253.9	8.39	No apical meristem protein, putative, expressed

MaNAC053	Ma0438520.1	288	32776.3	6.85	Transcription factor JUNGBRUNNEN 1
MaNAC054	Ma0500440.1	275	31401.1	8.2	No apical meristem protein, putative, expressed
MaNAC055	Ma0502120.1	309	34899.9	6.92	Uncharacterized protein
MaNAC056	Ma0503210.1	333	37159.4	5.16	No apical meristem protein, putative, expressed
MaNAC057	Ma0507350.1	314	35366.2	6.26	NAC domain-containing protein 68
MaNAC058	Ma0507360.1	354	39843.8	6.88	Putative NAC domain-containing protein 100
MaNAC059	Ma0516520.1	291	32633.2	5.64	No apical meristem protein, putative, expressed
MaNAC060	Ma0517940.1	283	32185.6	7.38	Uncharacterized protein
MaNAC061	Ma0520040.1	333	37746.1	7.79	NAC domain-containing protein 86
MaNAC062	Ma0520080.1	262	30464.6	8.45	Putative NAC domain-containing protein 102
MaNAC063	Ma0520400.1	368	40885.6	5.31	No apical meristem protein, putative, expressed
MaNAC064	Ma0521000.1	274	31045.8	6.42	Putative NAC domain-containing protein 21/22
MaNAC065	Ma0521450.1	245	28184.5	6.69	No apical meristem protein, putative, expressed
MaNAC066	Ma0525980.1	481	53500.6	6.72	Putative NAC domain-containing protein 74
MaNAC067	Ma0529000.1	300	33339.5	8.28	NAC domain-containing protein 21/22
MaNAC068	Ma0603480.1	304	33684.8	6.52	No apical meristem protein, putative, expressed
MaNAC069	Ma0604360.1	408	46203.1	8.05	No apical meristem protein, putative, expressed
MaNAC070	Ma0604820.1	468	51702	7.09	Putative NAC domain-containing protein 74
MaNAC071	Ma0607110.1	428	48189.6	8.04	No apical meristem protein, putative, expressed
MaNAC072	Ma0610180.1	348	39471.4	7.02	NAC domain-containing protein 100
MaNAC073	Ma0612700.1	1031	114290	5.84	Hypothetical protein
MaNAC074	Ma0618050.1	248	27635.8	8.67	Uncharacterized protein
MaNAC075	Ma0619050.1	217	23865.3	5.22	Putative NAC transcription factor ONAC010
MaNAC076	Ma0619100.1	291	32509.5	6.85	NAC domain-containing protein 2
MaNAC077	Ma0619970.1	193	21424	5.28	Putative NAC transcription factor NAM-B1
MaNAC078	Ma0620050.1	275	30882.9	9.06	NAC domain-containing protein 48
MaNAC079	Ma0620170.1	258	29077.5	8.36	Uncharacterized protein
MaNAC080	Ma0620690.1	246	27559.5	7.02	No apical meristem protein, putative, expressed
MaNAC081	Ma0622210.1	239	27204.5	8.7	NAC domain-containing protein 83
MaNAC082	Ma0624750.1	572	64218	4.55	Uncharacterized protein
MaNAC083	Ma0625140.1	236	26677.8	8.48	Uncharacterized protein
MaNAC084	Ma0625530.1	151	17517.29	10.29	Putative NAC domain-containing protein 2
MaNAC085	Ma0627170.1	275	30967.9	8.35	NAC domain-containing protein 2
MaNAC086	Ma0627580.1	198	21930.3	4.88	Putative NAC transcription factor 29
MaNAC087	Ma0628730.1	284	31283	8.8	Putative NAC domain-containing protein 8
MaNAC088	Ma0630630.1	320	35723.5	7.27	Putative Protein BEARSKIN2
MaNAC089	Ma0631760.1	286	31594.6	7.66	Putative NAC domain-containing protein 21/22
MaNAC090	Ma0632230.1	326	36332.2	8.37	NAC domain-containing protein 100
MaNAC091	Ma0633040.1	299	33111.6	8.77	NAC domain-containing protein 21/22
MaNAC092	Ma0633280.1	298	33925.8	6.89	No apical meristem protein, putative, expressed
MaNAC093	Ma0633960.1	347	38469.7	5.13	NAC domain-containing protein 100
MaNAC094	Ma0633980.1	346	39369.8	7.7	NAC transcription factor 29
MaNAC095	Ma0633990.1	271	31081.9	6.63	NAC domain-containing protein 68
MaNAC096	Ma0638460.1	279	31665.8	5.51	Putative NAC domain-containing protein 21/22
MaNAC097	Ma0638820.1	335	38720.3	6.67	NAC domain-containing protein 7
MaNAC098	Ma0700860.1	258	29034.6	7.24	Uncharacterized protein
MaNAC099	Ma0704130.1	293	33005	4.79	No apical meristem protein, putative, expressed
MaNAC100	Ma0706080.1	349	38988.3	7.98	NAC domain-containing protein 43
MaNAC101	Ma0706730.1	335	39179.7	6.46	NAC domain-containing protein 7
MaNAC102	Ma0709340.1	422	47822.4	7.36	No apical meristem protein, putative, expressed
MaNAC103	Ma0711300.1	318	35930.1	7.01	Putative NAC domain-containing protein 100
MaNAC104	Ma0719650.1	309	34651.2	7.93	NAC domain-containing protein 100
MaNAC105	Ma0721160.1	317	35187.7	8.31	No apical meristem protein, putative, expressed
MaNAC106	Ma0723270.1	286	32115.8	7.25	Uncharacterized protein
MaNAC107	Ma0723280.1	299	34147.1	6.17	NAC domain-containing protein 68
MaNAC108	Ma0723960.1	247	27401.8	9.08	No apical meristem protein, putative, expressed
MaNAC109	Ma0724640.1	279	31898.5	6.71	NAC domain-containing protein 30

MaNAC110	Ma0724710.1	306	34211.4	9.01	NAC domain-containing protein 48
MaNAC111	Ma0724800.1	409	45728.7	4.35	Putative NAC domain-containing protein 82
MaNAC112	Ma0725080.1	345	38848.2	8.91	Uncharacterized protein
MaNAC113	Ma0727560.1	406	45224.3	4.72	No apical meristem protein, putative, expressed
MaNAC114	Ma0801310.1	236	26928.7	9.6	NAC domain-containing protein 90
MaNAC115	Ma0806900.1	302	33446.9	9.64	No apical meristem protein, putative, expressed
MaNAC116	Ma0809680.1	170	18953.4	7.86	Putative NAC domain-containing protein 68
MaNAC117	Ma0811340.1	330	37941.4	7.01	NAC domain-containing protein 7
MaNAC118	Ma0811850.1	330	37760.4	4.98	NAC domain-containing protein 37
MaNAC119	Ma0813840.1	608	68308	5.48	Putative NAC domain-containing protein 86
MaNAC120	Ma0814960.1	274	30949.5	5.88	No apical meristem protein, putative, expressed
MaNAC121	Ma0817890.1	289	32046.1	8.8	Uncharacterized protein
MaNAC122	Ma0822240.1	280	31386.5	6.31	No apical meristem protein, putative, expressed
MaNAC123	Ma0825020.1	590	65340.4	4.45	Putative NAC domain-containing protein 16
MaNAC124	Ma0825500.1	302	33417.5	7.71	No apical meristem protein, putative, expressed
MaNAC125	Ma0833630.1	284	31562.5	8.07	No apical meristem protein, putative, expressed
MaNAC126	Ma0900570.1	291	32295.3	9.22	No apical meristem protein, putative, expressed
MaNAC127	Ma0901160.1	220	24981.3	10.25	NAC domain-containing protein 41
MaNAC128	Ma0901850.1	397	44460.5	6.16	Putative NAC domain-containing protein 8
MaNAC129	Ma0904890.1	285	32349.3	8.98	NAC transcription factor 29
MaNAC130	Ma0909630.1	305	35060.9	5.79	Putative NAC domain-containing protein 100
MaNAC131	Ma0909760.1	290	32137.4	8.47	Putative NAC domain-containing protein 21/22
MaNAC132	Ma0913900.1	237	27056.5	9.37	Putative NAC transcription factor 25
MaNAC133	Ma0917040.1	320	35992.5	6.95	NAC domain-containing protein 100
MaNAC134	Ma0918740.1	318	35960.3	5.23	CUP-SHAPED COTYLEDON3, putative, expressed
MaNAC135	Ma0919410.1	422	45913.6	8.32	Putative NAC transcription factor NAM-B2
MaNAC136	Ma0920430.1	685	75370.8	4.41	No apical meristem protein, putative, expressed
MaNAC137	Ma0924060.1	658	72711.2	4.5	No apical meristem protein, putative, expressed
MaNAC138	Ma0924410.1	246	27982.1	5.22	NAC domain-containing protein 45
MaNAC139	Ma0924910.1	356	40312	6.79	NAC domain-containing protein 43
MaNAC140	Ma0926680.1	303	33772.3	7.82	NAC domain-containing protein 21/22
MaNAC141	Ma0928160.1	336	37680.8	8.7	NAC domain-containing protein 100
MaNAC142	Ma0930350.1	244	27453.6	9.62	NAC domain-containing protein 90
MaNAC143	Ma1003880.1	496	53401.8	4.34	Hypothetical protein
MaNAC144	Ma1003900.1	435	46539.3	4.24	Hypothetical protein
MaNAC145	Ma1004160.1	334	37704	7.85	Uncharacterized protein
MaNAC146	Ma1004360.1	380	42916.9	4.59	Putative NAC domain-containing protein 16
MaNAC147	Ma1004400.1	292	32039.7	8.43	NAC domain-containing protein 48
MaNAC148	Ma1004550.1	248	27883.9	7.3	Putative NAC domain-containing protein 61
MaNAC149	Ma1004910.1	331	37405.1	7.02	Putative NAC domain-containing protein 100
MaNAC150	Ma1009160.1	332	37296.1	6.78	Uncharacterized protein
MaNAC151	Ma1009450.1	268	30563.4	8.2	Uncharacterized protein
MaNAC152	Ma1011090.1	207	22816.9	6.11	Putative NAC transcription factor NAM-1
MaNAC153	Ma1012120.1	242	26793	8.85	No apical meristem protein, putative, expressed
MaNAC154	Ma1013020.1	303	34543.5	9.87	Uncharacterized protein
MaNAC155	Ma1014520.1	312	35778.5	8.46	Protein SOMBRERO
MaNAC156	Ma1016990.1	219	24835.9	7.97	Putative NAC transcription factor NAM-2
MaNAC157	Ma1019880.1	321	35536.8	7.34	No apical meristem protein, putative, expressed
MaNAC158	Ma1021690.1	274	31795	9.09	Protein SOMBRERO
MaNAC159	Ma1022290.1	356	38456.1	8.45	Protein CUP-SHAPED COTYLEDON 2
MaNAC160	Ma1026130.1	333	36520.1	8.48	Putative NAC domain-containing protein 59
MaNAC161	Ma1027550.1	250	28451.8	9.14	No apical meristem protein, putative, expressed
MaNAC162	Ma1029220.1	300	34191.2	8.41	NAC domain-containing protein 68
MaNAC163	Ma1100790.1	279	31267.1	5.62	No apical meristem protein, putative, expressed
MaNAC164	Ma1101240.1	263	30153	9.41	No apical meristem protein, putative, expressed
MaNAC165	Ma1101340.1	393	44308.1	4.91	NAC domain-containing protein 86
MaNAC166	Ma1102940.1	330	37836.4	5.35	NAC domain-containing protein 37

MaNAC167	Ma1103600.1	311	35226.4	7.09	Putative NAC domain-containing protein 7
MaNAC168	Ma1105590.1	254	28578.5	11	Putative Transcription factor (Fragment)
MaNAC169	Ma1107480.1	713	79544.1	6.81	Uncharacterized protein
MaNAC170	Ma1108540.1	300	33853.6	9.18	Putative Transcription factor JUNGBRUNNEN 1
MaNAC171	Ma1108570.1	439	49301.8	4.26	No apical meristem protein, putative, expressed
MaNAC172	Ma1115400.1	589	66648.9	7.25	Putative NAC domain-containing protein 45
MaNAC173	Ma1116010.1	337	38921.3	6.45	NAC domain-containing protein 7
MaNAC174	Ma1116350.1	371	41078.7	5.75	No apical meristem protein, putative, expressed
MaNAC175	Ma1120940.1	202	23327.6	5.67	Uncharacterized protein
MaNAC176	Ma1121100.1	263	29588.1	5.05	No apical meristem protein, putative, expressed
MaNAC177	Ma1124060.1	306	33992.1	7.82	Putative NAC domain-containing protein 21/22
MaNAC178	Ma1124870.1	319	36449.6	7.32	No apical meristem protein, putative, expressed
MaNAC179	Ma0101430.1	457	48763.5	4.28	Hypothetical protein
MaNAC180	Ma0001720.1	381	42594.4	5.06	NAC domain-containing protein 43
MaNAC181	Ma0002930.1	399	44822.2	5.83	Putative NAC domain-containing protein 8

Table S2. Segmentally duplicated MaNAC gene pairs.

Gene ID	Gene ID
MaNAC181	MaNAC128
MaNAC004	MaNAC059
MaNAC019	MaNAC155
MaNAC041	MaNAC107
MaNAC043	MaNAC113
MaNAC074	MaNAC153
MaNAC107	MaNAC162
MaNAC118	MaNAC166
MaNAC081	MaNAC127
MaNAC089	MaNAC091
MaNAC091	MaNAC140
MaNAC105	MaNAC124
MaNAC146	MaNAC171
MaNAC064	MaNAC163
MaNAC011	MaNAC136
MaNAC041	MaNAC044
MaNAC091	MaNAC131

Table S3. Collinear MaNAC gene pairs.

Gene ID	Gene ID
MaNAC093	MaNAC149
MaNAC093	MaNAC133
MaNAC058	MaNAC093
MaNAC094	MaNAC129
MaNAC094	MaNAC154
MaNAC034	MaNAC104
MaNAC034	MaNAC072
MaNAC042	MaNAC087
MaNAC016	MaNAC087
MaNAC031	MaNAC165
MaNAC033	MaNAC154
MaNAC122	MaNAC133
MaNAC058	MaNAC122
MaNAC027	MaNAC122
MaNAC072	MaNAC122
MaNAC024	MaNAC132
MaNAC121	MaNAC157
MaNAC126	MaNAC157
MaNAC040	MaNAC106
MaNAC159	MaNAC160
MaNAC114	MaNAC148
MaNAC080	MaNAC148
MaNAC142	MaNAC148
MaNAC047	MaNAC165
MaNAC119	MaNAC165
MaNAC145	MaNAC170
MaNAC032	MaNAC065
MaNAC065	MaNAC164
MaNAC052	MaNAC071
MaNAC071	MaNAC161
MaNAC071	MaNAC102
MaNAC091	MaNAC131
MaNAC131	MaNAC140
MaNAC089	MaNAC131
MaNAC064	MaNAC120
MaNAC120	MaNAC176
MaNAC120	MaNAC163
MaNAC012	MaNAC088
MaNAC019	MaNAC023
MaNAC023	MaNAC155
MaNAC105	MaNAC124
MaNAC068	MaNAC105
MaNAC115	MaNAC177
MaNAC039	MaNAC115
MaNAC067	MaNAC115
MaNAC039	MaNAC177
MaNAC067	MaNAC177
MaNAC146	MaNAC171
MaNAC011	MaNAC136
MaNAC018	MaNAC102
MaNAC050	MaNAC098
MaNAC050	MaNAC053
MaNAC064	MaNAC176

MaNAC048	MaNAC051
MaNAC017	MaNAC051
MaNAC078	MaNAC147
MaNAC035	MaNAC153
MaNAC035	MaNAC074
MaNAC035	MaNAC083
MaNAC055	MaNAC130
MaNAC092	MaNAC130
MaNAC091	MaNAC140
MaNAC089	MaNAC091
MaNAC017	MaNAC048
MaNAC074	MaNAC153
MaNAC083	MaNAC153
MaNAC078	MaNAC110
MaNAC006	MaNAC096
MaNAC165	MaNAC172
MaNAC075	MaNAC152
MaNAC055	MaNAC098
MaNAC053	MaNAC055
MaNAC055	MaNAC092
MaNAC053	MaNAC098
MaNAC041	MaNAC044
MaNAC041	MaNAC107
MaNAC074	MaNAC083
MaNAC010	MaNAC178
MaNAC052	MaNAC161
MaNAC052	MaNAC102
MaNAC089	MaNAC140
MaNAC117	MaNAC173
MaNAC117	MaNAC167
MaNAC072	MaNAC104
MaNAC129	MaNAC154
MaNAC039	MaNAC067
MaNAC019	MaNAC158
MaNAC019	MaNAC155
MaNAC063	MaNAC174
MaNAC102	MaNAC161
MaNAC020	MaNAC156
MaNAC044	MaNAC107
MaNAC118	MaNAC166
MaNAC136	MaNAC137
MaNAC005	MaNAC032
MaNAC032	MaNAC099
MaNAC032	MaNAC164
MaNAC005	MaNAC099
MaNAC005	MaNAC164
MaNAC066	MaNAC070
MaNAC053	MaNAC092
MaNAC079	MaNAC109
MaNAC107	MaNAC162
MaNAC028	MaNAC100
MaNAC081	MaNAC127
MaNAC058	MaNAC133
MaNAC027	MaNAC133
MaNAC155	MaNAC158
MaNAC167	MaNAC173
MaNAC116	MaNAC168

MaNAC080	MaNAC142
MaNAC046	MaNAC164
MaNAC027	MaNAC058
MaNAC022	MaNAC099
MaNAC043	MaNAC113
MaNAC057	MaNAC107
MaNAC057	MaNAC095

Table S4. The parameters of selection pressure on segmentally duplicated gene pairs of the MaNAC family.

Gene ID	Gene ID	Ka	Ks	Ka/Ks	Divergence time (Mya)
MaNAC181	MaNAC128	0.141888	0.57179	0.248147	63.53222222
MaNAC004	MaNAC059	0.114398	0.605641	0.188888	67.29344444
MaNAC019	MaNAC155	0.0881073	0.736393	0.119647	81.82144444
MaNAC041	MaNAC107	0.105324	0.592176	0.17786	65.79733333
MaNAC043	MaNAC113	0.241341	0.508056	0.475029	56.45066667
MaNAC074	MaNAC153	0.095273	0.719969	0.132329	79.99655556
MaNAC107	MaNAC162	0.118983	0.617194	0.19278	68.57711111
MaNAC118	MaNAC166	0.0933115	0.581973	0.160336	64.66366667
MaNAC081	MaNAC127	0.160783	0.319079	0.503896	35.45322222
MaNAC089	MaNAC091	0.168851	0.468952	0.360061	52.10577778
MaNAC091	MaNAC140	0.104425	0.404794	0.25797	44.97711111
MaNAC105	MaNAC124	0.0999103	0.801044	0.124725	89.00488889
MaNAC146	MaNAC171	0.191449	0.475252	0.402838	52.80577778
MaNAC064	MaNAC163	0.139041	0.61259	0.226972	68.06555556
MaNAC011	MaNAC136	0.182734	0.432632	0.422377	48.07022222
MaNAC041	MaNAC044	0.12302	0.565754	0.217445	62.86155556
MaNAC091	MaNAC131	0.144529	0.317578	0.455098	35.28644444

Mya, million years ago

Table S5. Reported NAC genes from *Musa acuminate* in literatures (E represents ethylene, the number indicates the days after the treatment).

Gene symbol	0-pulp	E-1-pulp	E-3-pulp	E-5-pulp	0-peel	E-1-peel	E-3-peel	E-5-peel
MaNAC001	0.00	0.00	0.02	0.17	0.00	0.02	0.00	0.05
MaNAC002	0.00	0.00	0.00	0.00	0.00	0.00	0.00	0.00
MaNAC003	4.47	0.57	0.22	0.13	8.54	0.87	0.89	2.51
MaNAC004	0.00	0.00	0.00	0.00	0.00	0.00	0.00	0.00
MaNAC005	0.00	0.00	0.00	0.00	0.00	0.00	0.00	0.00
MaNAC006	0.04	0.00	0.00	0.00	0.00	0.00	0.00	0.00
MaNAC007	0.00	0.00	0.00	0.00	0.00	0.00	0.00	0.00
MaNAC008	1.92	1.43	0.88	1.71	0.10	0.16	0.59	3.68
MaNAC009	10.77	13.92	12.58	10.18	15.83	92.85	129.29	105.25
MaNAC010	0.00	0.00	0.00	0.00	0.06	0.02	0.07	0.02
MaNAC011	5.18	7.78	8.90	11.01	9.01	6.97	9.84	9.56
MaNAC012	0.00	0.00	0.00	0.00	0.00	0.00	0.00	0.00
MaNAC013	0.57	1.54	0.82	0.51	1.91	1.35	1.14	1.89
MaNAC014	0.00	0.00	0.00	0.00	0.00	0.00	0.00	0.00
MaNAC015	0.00	0.00	0.00	0.00	0.00	0.00	0.00	0.00
MaNAC016	8.41	16.82	157.12	237.05	1.08	29.13	142.07	130.64
MaNAC017	0.07	0.05	0.03	0.03	0.18	0.01	0.01	0.10
MaNAC018	0.02	0.00	0.01	0.01	0.01	0.01	0.02	0.01
MaNAC019	0.00	0.00	0.03	0.00	0.00	0.00	0.00	0.00
MaNAC020	6.74	2.62	1.09	0.96	7.01	6.99	2.30	1.48
MaNAC021	0.51	1.36	1.87	2.42	2.67	1.39	1.34	2.42
MaNAC022	0.00	0.00	0.00	0.00	0.03	0.21	0.00	0.00
MaNAC023	0.00	0.00	0.00	0.00	0.00	0.00	0.00	0.00
MaNAC024	0.00	0.00	0.00	0.00	0.00	0.00	0.00	0.00
MaNAC025	0.60	0.90	3.33	3.17	1.93	2.76	2.98	7.41
MaNAC026	0.00	0.07	0.13	0.63	0.06	0.00	0.00	0.00
MaNAC027	5.90	20.35	5.62	2.43	2.88	24.94	12.22	17.60
MaNAC028	0.00	0.01	0.00	0.00	0.00	0.00	0.00	0.00
MaNAC029	1.67	2.00	3.61	4.18	4.84	3.29	2.00	4.59
MaNAC030	20.42	17.35	7.31	7.21	14.87	6.31	7.59	6.33
MaNAC031	0.00	0.00	0.00	0.00	0.00	0.00	0.00	0.00
MaNAC032	0.00	0.00	0.00	0.00	0.00	0.34	0.00	0.00
MaNAC033	177.08	63.16	125.05	90.55	34.46	91.77	120.60	83.55
MaNAC034	0.00	0.00	0.00	0.04	0.00	0.02	0.14	0.10
MaNAC035	2.28	2.68	4.53	4.30	4.42	8.00	3.91	1.47
MaNAC036	0.32	0.82	1.17	2.10	0.74	9.52	5.26	15.45
MaNAC037	0.00	0.00	0.00	0.00	0.30	0.58	0.30	0.38
MaNAC038	0.00	0.00	0.00	0.06	0.00	0.00	0.09	0.02
MaNAC039	0.00	0.03	0.07	0.02	0.06	0.75	1.43	2.63
MaNAC040	19.58	8.69	9.72	5.66	30.52	64.26	72.91	43.85
MaNAC041	1.09	6.22	1.04	0.36	3.04	4.06	0.20	0.40
MaNAC042	0.00	0.00	0.00	0.00	0.00	0.00	0.04	0.00
MaNAC043	0.23	1.63	2.64	4.25	2.65	2.62	2.26	5.13
MaNAC044	0.25	0.23	0.72	0.51	0.72	1.03	1.19	0.86
MaNAC045	0.19	1.23	2.39	0.29	0.09	0.34	0.18	0.15
MaNAC046	0.00	0.00	0.00	0.00	0.60	0.05	0.00	0.06
MaNAC047	0.13	0.00	0.00	0.00	0.00	0.00	0.00	0.00

MaNAC048	1.29	1.26	2.15	3.52	3.49	1.83	2.66	6.04
MaNAC049	0.00	0.00	0.00	0.00	0.00	0.00	0.00	0.00
MaNAC050	0.00	0.00	0.00	0.00	0.00	0.00	0.00	0.00
MaNAC051	2.14	3.59	5.30	5.77	6.86	4.89	6.98	13.12
MaNAC052	0.00	0.01	0.00	0.00	0.07	0.01	0.00	0.00
MaNAC053	0.80	1.33	0.29	0.33	0.43	0.35	0.24	0.34
MaNAC054	0.00	0.11	0.46	0.44	0.36	2.44	3.38	2.05
MaNAC055	0.00	0.00	0.00	0.00	0.20	0.06	0.00	0.00
MaNAC056	0.00	0.18	0.30	0.20	0.01	0.04	0.10	0.20
MaNAC057	1.32	0.74	0.37	0.67	5.14	1.43	0.23	1.82
MaNAC058	47.30	39.92	12.20	13.96	20.43	38.01	22.86	27.65
MaNAC059	0.00	0.00	0.00	0.04	0.03	0.00	0.00	0.00
MaNAC060	66.86	13.29	1.70	0.55	13.67	8.84	5.18	6.36
MaNAC061	0.26	0.31	0.24	0.28	0.08	0.04	0.05	0.08
MaNAC062	0.28	0.02	0.00	0.00	0.14	0.00	0.00	0.00
MaNAC063	0.25	0.02	0.00	0.01	0.27	0.02	0.08	0.11
MaNAC064	2.24	1.27	0.35	0.39	2.30	0.20	0.04	0.06
MaNAC065	0.07	0.08	0.05	0.05	1.14	0.30	0.10	0.28
MaNAC066	0.97	1.30	1.57	2.16	6.30	2.66	1.85	3.73
MaNAC067	1.96	2.05	1.41	0.30	1.53	1.74	2.76	2.39
MaNAC068	1.85	15.65	5.86	1.50	2.68	15.04	1.77	4.29
MaNAC069	0.00	0.00	0.00	0.00	0.00	0.00	0.00	0.00
MaNAC070	1.49	2.52	3.80	5.52	3.51	3.23	2.59	3.77
MaNAC071	0.08	0.00	0.05	0.01	0.01	0.01	0.02	0.11
MaNAC072	0.00	0.03	0.00	0.05	0.16	0.51	0.15	0.16
MaNAC073	0.14	0.16	0.50	0.59	0.43	0.22	0.41	1.14
MaNAC074	33.65	127.45	159.91	198.63	31.46	170.31	116.95	86.65
MaNAC075	117.74	3.69	0.86	0.30	40.52	3.21	3.32	1.68
MaNAC076	0.96	0.64	8.27	12.81	0.39	0.71	0.85	1.59
MaNAC077	0.33	0.00	0.00	0.00	0.06	0.00	0.00	0.00
MaNAC078	82.54	52.29	9.76	15.78	59.27	136.95	35.12	32.48
MaNAC079	0.00	0.00	0.00	0.00	0.00	0.00	0.00	0.00
MaNAC080	0.00	0.04	0.00	0.05	0.08	0.00	0.00	0.00
MaNAC081	0.13	1.03	0.02	0.00	0.24	0.07	0.00	0.00
MaNAC082	12.41	9.45	7.38	12.28	15.63	11.39	13.49	12.97
MaNAC083	31.84	10.31	185.94	298.85	3.25	11.02	62.87	34.92
MaNAC084	0.14	23.34	13.05	5.11	0.20	7.05	14.57	12.70
MaNAC085	107.84	19.09	17.25	18.38	11.16	0.96	0.14	1.54
MaNAC086	20.78	1.45	0.08	0.08	3.09	1.83	0.06	0.00
MaNAC087	4.34	8.13	7.08	8.28	0.18	4.64	12.18	9.87
MaNAC088	0.04	0.00	0.05	0.05	0.01	0.06	0.00	0.02
MaNAC089	0.53	0.11	0.08	0.00	0.57	0.12	0.03	0.00
MaNAC090	1.12	0.38	0.00	0.00	6.42	0.18	0.00	0.00
MaNAC091	1.93	1.84	0.52	0.11	1.99	4.39	1.80	0.84
MaNAC092	0.00	0.21	5.47	5.65	0.14	15.44	19.52	20.40
MaNAC093	0.55	0.24	0.15	0.11	0.21	0.15	0.10	0.21
MaNAC094	4.38	231.70	1190.96	1402.10	31.76	735.50	704.54	926.00
MaNAC095	34.36	123.06	419.77	560.32	48.69	382.70	630.08	455.56
MaNAC096	0.00	0.02	0.00	0.00	0.00	0.02	0.00	0.00
MaNAC097	0.00	0.00	0.00	0.00	0.00	0.00	0.00	0.00
MaNAC098	0.21	0.06	0.02	0.03	6.86	1.18	0.57	0.87

MaNAC099	0.00	0.00	0.13	0.48	0.04	0.00	0.00	0.12
MaNAC100	0.22	0.01	0.02	0.00	1.02	0.03	0.00	0.00
MaNAC101	0.00	0.00	0.00	0.00	0.00	0.00	0.00	0.00
MaNAC102	0.00	0.00	0.00	0.00	0.02	0.02	0.00	0.01
MaNAC103	0.25	0.62	26.79	43.70	4.20	1.29	6.73	9.07
MaNAC104	0.02	0.12	0.14	0.10	0.32	1.03	0.83	0.62
MaNAC105	0.02	0.01	0.05	0.02	0.02	0.05	0.02	0.15
MaNAC106	220.85	81.32	42.66	14.92	25.77	27.45	54.85	16.02
MaNAC107	13.07	41.41	13.52	5.16	48.63	38.88	4.94	8.08
MaNAC108	2.46	2.61	1.16	0.35	2.94	4.25	1.54	0.41
MaNAC109	0.05	0.15	0.20	0.29	0.00	0.00	0.16	0.07
MaNAC110	1301.78	294.90	36.46	16.41	83.06	194.49	113.26	55.50
MaNAC111	195.96	84.03	34.88	35.45	389.95	72.11	76.80	129.86
MaNAC112	0.00	0.00	0.00	0.00	1.16	2.45	1.69	1.65
MaNAC113	0.47	4.56	7.65	3.87	0.65	14.90	26.44	33.30
MaNAC114	0.00	0.03	0.00	0.00	0.09	0.00	0.00	0.06
MaNAC115	0.64	0.90	0.55	0.78	0.86	3.13	6.17	6.52
MaNAC116	2.47	0.00	0.12	0.52	0.13	0.00	0.00	0.00
MaNAC117	0.00	0.00	0.02	0.00	0.00	0.00	0.05	0.01
MaNAC118	0.28	0.09	0.00	0.02	0.14	0.00	0.14	0.02
MaNAC119	0.08	0.01	0.05	0.05	0.00	0.07	0.06	0.03
MaNAC120	0.09	0.01	0.00	0.00	1.46	0.05	0.00	0.00
MaNAC121	0.00	0.00	0.00	0.00	0.00	0.00	0.00	0.00
MaNAC122	3.79	2.52	0.70	0.94	4.28	13.36	8.00	11.18
MaNAC123	19.27	22.05	15.99	16.78	15.45	28.13	28.12	24.30
MaNAC124	0.00	0.00	0.20	0.17	0.00	0.02	0.49	0.74
MaNAC125	0.34	0.36	0.04	0.00	6.46	6.37	2.19	1.98
MaNAC126	0.00	0.00	0.00	0.00	0.00	0.00	0.00	0.00
MaNAC127	49.99	59.09	1.69	0.55	23.30	100.84	15.02	12.36
MaNAC128	0.02	0.00	0.00	0.01	0.06	0.02	0.10	0.08
MaNAC129	312.58	334.86	890.71	889.67	30.72	757.93	1449.98	937.38
MaNAC130	0.00	0.00	0.14	0.40	0.08	0.04	0.02	0.08
MaNAC131	174.54	537.14	137.41	74.36	46.11	520.61	275.32	158.75
MaNAC132	0.00	0.00	0.00	0.00	0.00	0.00	0.00	0.00
MaNAC133	10.07	5.50	5.18	4.47	1.37	2.88	8.48	10.13
MaNAC134	0.00	0.00	0.00	0.00	0.00	0.00	0.00	0.00
MaNAC135	0.86	0.03	0.01	0.02	0.03	0.00	0.00	0.00
MaNAC136	4.05	8.56	19.26	29.43	11.88	5.97	11.16	13.55
MaNAC137	21.15	40.31	28.59	21.41	38.10	24.81	23.55	18.61
MaNAC138	0.00	0.00	0.00	0.00	0.00	0.00	0.00	0.00
MaNAC139	5.65	0.44	0.56	0.83	0.40	0.13	0.20	0.34
MaNAC140	56.87	23.62	22.63	12.23	20.54	17.69	19.23	9.93
MaNAC141	18.85	1.43	0.05	0.05	4.28	0.52	0.08	0.00
MaNAC142	0.00	0.12	0.00	0.17	0.11	0.07	0.00	0.00
MaNAC143	0.00	0.00	0.00	0.00	0.00	0.00	0.00	0.00
MaNAC144	0.00	0.00	0.00	0.00	0.00	0.00	0.00	0.00
MaNAC145	0.00	0.00	0.00	0.00	0.92	0.02	0.01	0.01
MaNAC146	10.04	10.27	6.82	8.38	7.32	5.88	7.15	6.93
MaNAC147	28.62	1.77	3.98	10.42	29.09	1.93	1.43	1.92
MaNAC148	0.00	0.00	0.00	0.00	3.03	0.16	0.30	0.81

MaNAC149	0.14	0.31	0.49	0.34	0.00	3.26	11.02	14.69
MaNAC150	0.00	0.01	0.12	0.09	0.00	0.00	0.00	0.04
MaNAC151	0.00	0.00	0.02	0.00	0.10	0.11	0.07	0.00
MaNAC152	0.00	0.09	0.11	0.00	0.19	0.92	4.65	2.82
MaNAC153	384.27	164.13	17.39	8.48	137.98	27.66	4.65	9.51
MaNAC154	11.46	5.76	3.37	1.85	7.69	38.35	10.82	8.88
MaNAC155	0.00	0.00	0.00	0.00	0.00	0.00	0.00	0.00
MaNAC156	16.74	8.25	2.07	1.21	41.82	37.90	10.34	13.59
MaNAC157	0.00	0.00	0.66	0.90	0.04	0.00	0.07	0.07
MaNAC158	0.00	0.00	0.00	0.00	0.03	0.00	0.07	0.00
MaNAC159	0.09	0.00	0.00	0.00	0.00	0.00	0.00	0.00
MaNAC160	0.00	0.00	0.00	0.00	0.00	0.00	0.00	0.00
MaNAC161	0.00	0.00	0.03	0.00	0.02	0.02	0.00	0.04
MaNAC162	0.36	0.71	0.29	0.24	2.16	0.64	0.20	0.99
MaNAC163	0.17	0.15	0.06	0.12	1.69	0.05	0.07	0.02
MaNAC164	4.40	0.73	0.04	0.02	5.82	0.62	0.25	0.42
MaNAC165	0.00	0.00	0.00	0.00	0.00	0.00	0.00	0.00
MaNAC166	0.05	0.03	0.00	0.04	0.00	0.02	0.03	0.00
MaNAC167	0.00	0.00	0.00	0.00	0.00	0.00	0.00	0.00
MaNAC168	0.00	0.00	0.00	0.00	0.00	0.00	0.00	0.00
MaNAC169	1.62	1.96	8.06	10.82	2.29	2.17	3.94	5.81
MaNAC170	0.00	0.00	0.00	0.00	0.00	0.00	0.00	0.00
MaNAC171	4.09	13.11	13.91	15.58	10.78	8.18	12.54	11.73
MaNAC172	0.00	0.00	0.00	0.00	0.00	0.00	0.00	0.00
MaNAC173	0.09	0.07	0.11	0.02	0.07	0.02	0.01	0.01
MaNAC174	0.25	0.19	1.12	1.71	0.23	0.59	0.18	0.11
MaNAC175	0.00	0.03	0.63	0.03	0.12	0.00	0.00	0.00
MaNAC176	6.13	2.48	0.62	0.24	0.07	0.00	0.00	0.00
MaNAC177	6.90	2.25	0.12	0.02	15.90	9.72	1.66	6.14
MaNAC178	0.18	0.55	0.70	1.27	0.34	2.89	2.55	5.60
MaNAC179	27.53	11.53	2.24	3.21	18.49	9.26	14.52	16.98
MaNAC180	0.19	0.00	0.00	0.00	0.00	0.00	0.00	0.00
MaNAC181	0.00	0.00	0.01	0.04	0.08	0.05	0.00	0.02

Table S6. The primer sequences used for RT-PCR.

Genes	Primer sequence (5′ to 3′)
MaNAC094	F: GCAACCATCTACACCACCGA
	R: GCTGATTGAAGAAGCGCTGG
MaNAC129	F: CAAGGGGAGCAAGACGAACT
	R: TTGTAGCAGTTGCTCCTCCG
MaNAC009	F: CGAAGCTCTGAGGAGCAACA
	R: TAGATTCGGCAGAGGACCCA
MaNAC095	F: TTCCCGGAGGTGGATGATCT
	R: AAGTCCACGAACCAATCGCT
MaNAC074	F: ATCACGTCTTCCTGGTGCAG
	R: GCTTTCCCCGGTAGAAGACC
MaNAC103	F: GGTAGGGGACGAGTGGTACT
	R: GGTGGCTCTGTTTGTCCTCA
MaNAC083	F: GAAAGTCTTCTGCTGCCCCT
	R: CCCATGGATCGAGTTTTGCG
MaNAC035	F: CGAAGAGCTCGTGGTTCAGT
	R: AGGTCGAGGTCAGGGATGAA
MaNAC016	F: GCCCGAGAAGACAAACTGGA
	R: CCACCAACTCTCCGTCCTTC
MaNAC087	F: GCAGGCGTGAAGTTTGATCC

F, forward; R. reverse

Table S7. The primer sequences used for used for subcellular localization.

Genes	Primer sequence (5′ to 3′)
MaNAC094	F: GGGGATCCTCTAGAGTCGACATGTCGGATCCTGCGTCG
	R: CTCACCATCTGCAGGTCGACATGTAATCCCAGCTGAGG
MaNAC129	F: GGGGATCCTCTAGAGTCGACATGTCGGATCCCGCGTCT
	R: CTCACCATCTGCAGGTCGACCAGATGAGAGCCGGGCAG
MaNAC009	F: GGGGATCCTCTAGAGTCGACATGTCGAATCCGACCAGTCTTC
	R: CTCACCATCTGCAGGTCGACCATCTCGTATGCTGCTTTG
MaNAC095	F: GGGGATCCTCTAGAGTCGACATGGGGAGGAGGACGAGAGA
	R: CTCACCATCTGCAGGTCGACCATAGATGGGAAGAAGCAGC
MaNAC074	F: GGGGATCCTCTAGAGTCGACATGGACAGGAAGCCGAGTG
	R: CTCACCATCTGCAGGTCGACTGGAAGTGATCTGCTAGAGCTG
MaNAC103	F: GGGGATCCTCTAGAGTCGACATGCTCAGGTTGATGATCGAGG
	R: CTCACCATCTGCAGGTCGACGTAGTTCCATGACAGCACATCG
MaNAC083	F: GGGGATCCTCTAGAGTCGACATGGAGAGCAAGCCAAGCTT
	R: CTCACCATCTGCAGGTCGACGGGCTCACTTCCTTGTGG
MaNAC035	F: GGGGATCCTCTAGAGTCGACATGGACAACAAGCCACGCC
	R: CTCACCATCTGCAGGTCGACGCTGCCAGAACTGGTTTCC
MaNAC016	F: GGGGATCCTCTAGAGTCGACATGGTTTCCATCAGCAAGAAGTC
	R: CTCACCATCTGCAGGTCGACGGAAAGGAAGGGAGGAGG
MaNAC087	F: GGGGATCCTCTAGAGTCGACATGGTTCTAATCACCAAGAAATCAC

F, forward; R. reverse
